# Efficient Bayesian analysis of occupancy models with logit link functions

**DOI:** 10.1002/ece3.4850

**Published:** 2019-02-05

**Authors:** Allan E. Clark, Res Altwegg

**Affiliations:** ^1^ Department of Statistical Sciences University of Cape Town Cape Town South Africa; ^2^ Center for Statistics in Ecology, Environment and Conservation (SEEC) University of Cape Town Rondebosch South Africa

**Keywords:** Bayesian spatial occupancy model, imperfect detection, occupancy model, Rcppocc, restricted spatial regression

## Abstract

Occupancy models (*Ecology*, 2002; 83: 2248) were developed to infer the probability that a species under investigation occupies a site. Bayesian analysis of these models can be undertaken using statistical packages such as *WinBUGS*,* OpenBUGS*,* JAGS*, and more recently *Stan*, however, since these packages were not developed specifically to fit occupancy models, one often experiences long run times when undertaking an analysis. Bayesian spatial single‐season occupancy models can also be fit using the R package *stocc*. The approach assumes that the detection and occupancy regression effects are modeled using probit link functions. The use of the logistic link function, however, is algebraically more tractable and allows one to easily interpret the coefficient effects of an estimated model by using odds ratios, which is not easily done for a probit link function for models that do not include spatial random effects. We develop a Gibbs sampler to obtain posterior samples from the posterior distribution of the parameters of various occupancy models (nonspatial and spatial) when logit link functions are used to model the regression effects of the detection and occupancy processes. We apply our methods to data extracted from the 2nd Southern African Bird Atlas Project to produce a species distribution map of the Cape weaver (*Ploceus capensis*) and helmeted guineafowl (*Numida meleagris*) for South Africa. We found that the Gibbs sampling algorithm developed produces posterior samples that are identical to those obtained when using *JAGS* and *Stan* and that in certain cases the posterior chains mix much faster than those obtained when using *JAGS*,* stocc*, and *Stan*. Our algorithms are implemented in the R package, *Rcppocc*. The software is freely available and stored on GitHub (https://github.com/AllanClark/Rcppocc).

## 1 INTRODUCTION

Occupancy models are an important statistical technique that was developed to make use of detection/nondetection data to infer the probability that a species under investigation occupies a site. When an occupancy study is undertaken, $n_{s}$ sites are visited a number of times to estimate the occupancy probability ($\underset{\overset{¯}{}}{p}si$) and conditional detection probability (***p***) of a species associated with each site in a region. The method can be viewed as an extension of logistic regression and allows one to estimate the occupancy probability at sites where none of the species being investigated have been detected. The model is formulated hierarchically, using Bernoulli random variables to specify the occupancy and detection processes, respectively, which can be modeled using site‐specific and survey‐specific explanatory variables, respectively (MacKenzie et al., 2002). Johnson, Conn, Hooten, Ray, and Pond (2013) note that occupancy models produce “unbiased inference when occupancy observations at nearby units are conditionally independent given any available covariates” but stress that “spatial autocorrelation may lead to biases and overestimated precision” of regression effects. This observation has lead to the development of various models to account for spatial autocorrelation in ecological survey data (Aing, Halls, Oken, Dobrow, & Fieberg, 2011; Gardner, Lawler, Ver Hoef, Magoun, & Kellie, 2010; Hoeting, Leecaster, & Bowden, 2000; Hooten, Larsen, & Wikle, 2003) and have extensively been used to guide environmental monitoring and assessment programs globally.

A number of methods have been used to fit occupancy models to data. These include maximum likelihood (MacKenzie et al., 2002); penalized maximum likelihood (Hutchinson, Valente, Emerson, Betts, & Dietterich, 2015; Moreno & Lele, 2010), Bayesian methods that employ *WinBUGS*,* OpenBUGS*,* JAGS*, or *Stan* as well as approximate methods such as those developed by Clark, Altwegg, and Ormerod (2016). Recently Dorazio and Rodriguez (2012) and Johnson et al. (2013) developed Gibbs algorithms to obtain posterior samples for the parameters of a nonspatial and spatial single‐season occupancy (SSO) model, respectively. Both approaches assume that detection and occupancy processes are modeled using probit link functions, which enables the use of data augmentation (Tanner & Wong, 1987) to obtain closed form expressions of the conditional posterior distributions of the parameters of the occupancy model.

Given that the probit and logistic functions are very similar and only differ in respect of the tails of the functions, analysis undertaken using either of the functions should produce similar occupancy and conditional detection probabilities (Dorazio & Rodriguez, 2012). However, the use of the logistic link function is algebraically more tractable and allows one to easily interpret the coefficient effects of an estimated model by using odds ratios, which is not easily done for a probit link function. This observation is particularly true for the nonspatial SSO model since no spatial random effects are included in this model; however, when spatial random effects are included in the model, the interpretation of the regression effects can be difficult (Boehm, Reich, & Bandyopadhyay, 2013).

The paper commences with a brief discussion of the link between logistic regression and occupancy models. Thereafter, we discuss the formulation of various popular Bayesian spatial occupancy models and develop a Gibbs sampling algorithm for a particular spatial occupancy model when the regression effects of the occupancy and detection processes are modeled using logit link functions. Before concluding, we analyze two detection/nondetection data sets of South African bird species to illustrate the methods developed in the paper. An R package (*Rcppocc*) has been developed to fit SSO models using Gibbs sampling which can be obtained at: https://github.com/AllanClark/Rcppocc.

## 2 MATERIAL AND METHODS

### 2.1 Logistic regression and occupancy models

Assume that $n_{s}$ sites are surveyed a number of times and detection/nondetection data are collected at all sites. Denote the observed data as a ragged matrix $y\;=\;[y_{\mathrm{ij}}]$ where $y_{\mathrm{ij}}\;=\;1$ if the species under investigation has been observed at site i during survey j and $y_{\mathrm{ij}}\;=\;0$ otherwise. Let the vector z represents the true species occupancy at the sites considered such that $z_{i}\;=\;1$ if the species occupies site i and $z_{i}\;=\;0$ if it does not occupy site i. The SSO model can be represented using the following hierarchical model, $z_{i}|\psi_{i}∼\text{Bernoulli}\left(\psi_{i}\right)$, $y_{\mathrm{ij}}|z_{i},p_{\mathrm{ij}}∼\text{Bernoulli}\left(z_{i}p_{\mathrm{ij}}\right)$ for all sites $i\;=\;1,\;\ldots ,\;n_{s}$; for all surveys $j\;=\;1,\;\ldots ,\;V_{i}$ (Royle & Dorazio, 2008). The variable $\psi_{i}$ denotes the probability occupancy probability at site i, while $p_{\mathrm{ij}}\;=\;\text{Pr}\left(y_{\mathrm{ij}}\;=\;1|z_{i}\;=\;1\right)$ denotes the conditional probability of detecting the species during the *j*th survey of site i given that the species is present at site i. In what follows we assume that the conditional detection and occupancy regression effects (α and β) are modeled using logit link functions such that $\text{logit}\left(\psi_{i}\right)\;=\;x_{i}^{T}\beta$ and $\text{logit}\left(p_{\mathrm{ij}}\right)\;=\;w_{\mathrm{ij}}^{T}\alpha$, where $x_{i}^{T}$ and $w_{\mathrm{ij}}^{T}$ are row vectors in design matrices, X (occupancy) and W (detection), respectively (as defined in Clark et al., 2016).

**Figure 1:**
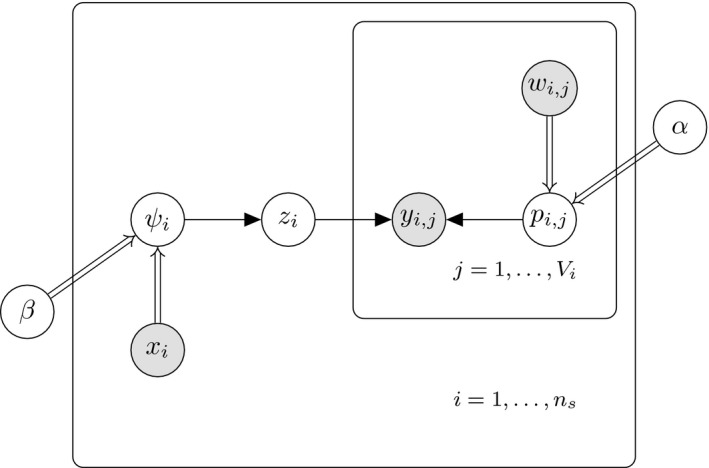
A directed acyclic graph illustrating the dependencies between the parameters and observed data for an SSO model. Shaded nodes represents observed data while all latent parameters are represented using unshaded nodes. Deterministic relationships are represented using double arrows while all stochastic relationships are represented using a single arrow

The joint posterior distribution of the parameters of the model is

$$\left[z,\alpha ,\beta |y\right]\propto \pi \left(\alpha \right)\pi \left(\beta \right)\left(\prod_{i=1}^{n_{s}}\psi_{i}^{z_{i}}{\left(1-\psi_{i}\right)}^{1-z_{i}}\right)\underset{\left\{i:z_{i}=1\right\}}{\prod_{i}\prod_{j}}p_{\mathrm{ij}}^{y_{\mathrm{ij}}}{\left(1-p_{\mathrm{ij}}\right)}^{1-y_{\mathrm{ij}}}$$

where π(α) and π(β) are the prior distributions of α and β, respectively. A directed acyclic graph of the above problem is displayed in Figure 1 below.

A Gibbs sampling algorithm for the parameters of this model requires sampling from [β|z], [α|z,y], and $\left[z_{i}\;=\;1|\alpha ,\beta ,y\right]$ for all sites where the species has not been observed. The first two conditional distributions have the following form,

(1) $$\begin{array}{cc} \left[\beta |z\right] & \propto \pi \left(\beta \right)\prod_{i=1}^{n_{s}}\psi_{i}^{z_{i}}{\left(1-\psi_{i}\right)}^{1-z_{i}}\;\text{and} \end{array}$$

(2) $$\begin{array}{cc} \left[\alpha |z,y\right] & \propto \pi \left(\alpha \right)\begin{array}{c} \prod_{i}\;\;\prod_{j}\\ \left\{i:z_{i}=1\right\} \end{array}p_{\mathrm{ij}}^{y_{\mathrm{ij}}}{\left(1-p_{\mathrm{ij}}\right)}^{1-y_{\mathrm{ij}}}. \end{array}$$

Notice that Equations [Link] and (1) are of the same form as the posterior distributions of the regression effects of a logistic regression model and therefore we adapt a Gibbs sampling scheme for logistic regression models to address the problem of obtaining posterior samples for the parameters of an occupancy model.

In a logistic regression context, Polson, Scott, and Windle (2013) show that posterior samples of the regression effects can be obtained by sampling from the conditional distributions of Pólya‐Gamma random variables and multivariate Gaussian distributions in turn. Their method is similar to that of Albert and Chib (1993) who developed a Gibbs algorithm to undertake probit regression, the only difference being that the sampling from truncated Gaussian distributions is replaced by sampling from Pólya‐Gamma distributions. The sampling methods developed by Polson et al. (2013) are exact since their Pólya‐Gamma sampling method is uniformly ergodic and converges to the correct posterior distribution (Choi, & Hobert, 2013).

For the SSO model, the conditional posterior distributions of $\beta |\omega_{\beta },y$ are derived by introducing Pólya‐Gamma latent variables, $\omega_{\beta }$, and noting that the contribution of the i^^{th}^ observation to a Bernoulli likelihood can be re‐expressed as

$$\psi_{i}^{z_{i}}{\left(1-\psi_{i}\right)}^{1-z_{i}}=\frac{\exp{\left(x_{i}^{T}\beta \right)}^{z_{i}}}{1+\exp\left(x_{i}^{T}\beta \right)}=\exp\left(\kappa_{i}x_{i}^{T}\beta \right)\int \exp\left(-\frac{\omega_{i,\beta }}{2}{\left(x_{i}^{T}\beta \right)}^{2}\right)p\left(\omega_{i,\beta }|1,0\right)d\omega_{i,\beta },$$

where $\kappa_{i}\;=\;z_{i}-0.5$ and $p(\omega_{i,\beta }|1,0)$ is the probability density function of a Pólya‐Gamma distribution with parameters 1 and 0 (Polson et al., 2013). The conditional posterior distribution of α is derived by using the same manipulation of the Bernoulli likelihood.

In the Supporting information (Appendix S1), we discuss the existing Gibbs algorithms used for undertaking logistic regression and demonstrate the use of the Pólya‐Gamma (PG) method by developing two Gibbs sampling algorithms for the parameters of SSO models. In Table 1, we summarize the Gibbs algorithms for an SSO model when using the PG method but provide the details regarding the algorithm in the Supporting Information (Appendix S2 and Appendix S3). We use the notation “a∼PG(b,c)” to indicate that the random variable a is a Pólya‐Gamma random variable with parameters b and c. Take note that the algorithm is identical to that developed by Dorazio and Rodriguez (2012) except that the sampling from truncated Gaussian distributions is replaced by sampling from Pólya‐Gamma distributions.

**Table 1:**
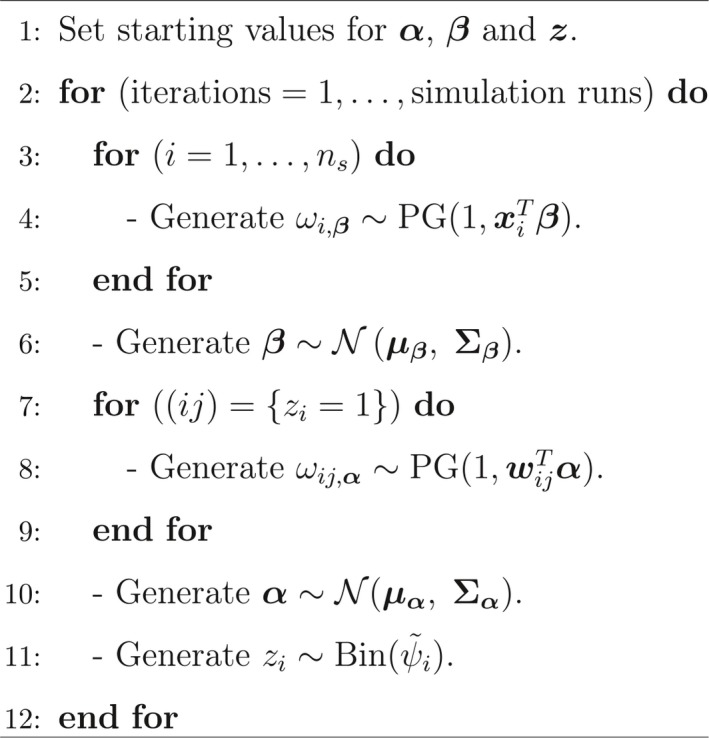
The Gibbs algorithm for undertaking a SSO model using the “PG” method (See the Supporting Information (Appendix S3) for the details pertaining to the parameter matrices of the conditional posterior distributions.)

### 2.2 Bayesian spatial SSO models

Spatial generalized linear mixed models (SGLMM) are an extension of the general linear model (Nelder & Wedderburn, 1972) that allows the link function of the expected value of the random variable under investigation to be modeled as a function of a spatial random variable/s. The formulation was first developed by Besag, York, and Mollié (1991) and has been extensively used in areas such as agriculture (Besag & Higdon, 1999), biostatistics (Gelfand & Vounatsou, 2003; Waller & Gotway, 2004), ecology (Lichstein, Simons, Shriner, & Franzreb, 2002) and species distribution modelling (Drouilly, Clark, & O'Riain, 2018; Gelfand et al., 2005; Hooten et al., 2003; Latimer, Wu, Gelfand, & Silander, 2006).

The paper by Gelfand et al. (2005) lead to the development of the R package *hSDM* (Vieilledent et al., 2014) in which the hSDM.siteocc.iCAR function can be used to fit a particular spatial occupancy model to detection/nondetection data. A region under investigation is subdivided into $n_{s}$ grid cells each which are surveyed a number of occasions. The model is formulated using Bernoulli latent random variables $z\;=\;{\left(z_{1},\ldots ,z_{n_{s}}\right)}^{T}$. Formally, $z_{i}|\psi_{i}∼\text{Bernoulli}\left(\psi_{i}\right)\;\text{with logit}\left(\psi_{i}\right)\;=\;x_{i}^{T}\beta \;+\;\rho_{i},\;\text{for all}\;i\;=\;1,\ldots ,n$, where $\rho \;=\;{\left(\rho_{1},\ldots ,\rho_{n_{s}}\right)}^{T}$ is a multivariate Gaussian random vector with mean 0 and correlation matrix defined using the neighborhood structure of the grid cells. The observation process is specified as in the nonspatial model. The documentation of the function indicates that posterior samples of the parameters of the model are obtained using the C programming language and utilizes an adaptive Metropolis algorithm (Metropolis, Rosenbluth, Rosenbluth, Teller, & Teller, 1953; Robert & Casella, 2013).

Johnson et al. (2013) develop two spatial occupancy models. They assume that probit link functions are used to model both the occupancy and detection processes and thereby rely on data augmentation to develop a Gibbs sampling algorithm to sample from the posterior distribution of the parameter of the models. For the probit case, the occupancy probability of a particular grid cell (for the standard occupancy model) is calculated as $\Phi \left(x_{i}^{T}\beta \right)\;=\;\text{Pr}\left(z_{i}\;=\;1\right)$. In a Bayesian context, such a probit model is formulated by defining a latent Gaussian random variable, ${\tilde{z}}_{i}$ with mean 0 and variance equal to 1 such that $\text{Pr}\left(z_{i}\;=\;1\right)\;=\;\text{Pr}\left({\tilde{z}}_{s}\;>\;0\right)$). In the first of their models, they allow ${\tilde{z}}_{s}$ to be spatially correlated such that ${\tilde{z}}_{i}\;=\;x_{i}^{T}\beta \;+\;\eta_{i}\;+\;\epsilon_{i}.$ $\beta \;=\;{\left(\eta_{1},\ldots ,\eta_{n_{s}}\right)}^{T}$ is defined as ρ above while $\epsilon_{i}∼N\left(0,1\right),\text{for all}i\;=\;1,\ldots ,n_{s}$.

Often the spatial random effects and fixed effects of a model are collinear when spatially varying covariates are included as fixed effects (Hanks, Schliep, Hooten, & Hoeting, 2015; Hodges & Reich, 2010; Hughes & Haran, 2013; Reich, Hodges, & Zadnik, 2006). The suggested solution to this problem was to include spatial random effects in the model specification that are orthogonal to the fixed effects and is known as *restricted spatial regression* (RSR). The second spatial model developed by Johnson et al. (2013) uses this method and redefines ${\tilde{z}}_{i}$ as ${\tilde{z}}_{i}\;=\;x_{i}^{T}\beta \;+\;k_{i}^{T}\underset{\overset{¯}{}}{t}heta\;+\;\epsilon_{i}$, where $k_{i}^{T}$ is a row vector of the design matrix K. The spatial random effects are modeled as

$$\begin{array}{cc} \theta |\tau & ∼N\left(0_{r},\frac{1}{\tau }{\left(K^{T}QK\right)}^{-1}\right)=N\left(0_{r},\frac{1}{\tau }M\right),\\ \tau & ∼G\left(i_{1},\;i_{2}\right)\;\;\text{and}\;\epsilon ∼N\left(0_{n},\;I_{n}\right). \end{array}$$

Q is a n×n ICAR precision matrix (Besag & Kooperberg, 1995) obtained using surveyed and unsurveyed locations, τ is a spatial precision parameter and $i_{1}$ and $i_{2}$ are known constants. Kelsall and Wakefield (1999) have suggested setting these parameters to 0.5 and 0.005, respectively, such that the prior mean of τ is 1,000. The matrix K consists of the first r (*r *≪ *n*) eigenvectors of $\omega \;=\;nRAR/1^{T}A1$ where $R\;=\;I_{n}-X{\left(X^{T}X\right)}^{-1}X^{T}$ and A is an association matrix with ${\left(ij\right)}^{\mathrm{th}}$ entry $A_{\mathrm{ij}}\;=\;1$ if sites i and j are neighbours and zero otherwise.

**Figure 2:**
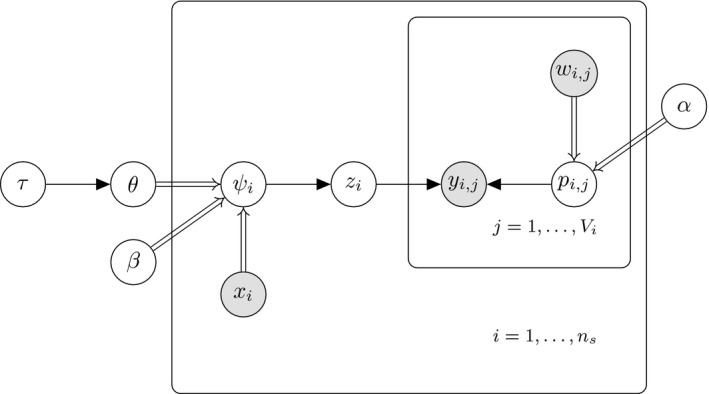
A directed acyclic graph illustrating the dependencies between the parameters and observed data for a spatial SSO model. Shaded nodes represents observed data while all latent parameters are represented using unshaded nodes. Deterministic relationships are represented using double arrows while all stochastic relationships are represented using a single arrow

In our formulation of the spatial occupancy model, we model the occupancy probabilities at all grid cells as

$$\text{logit}\left(\psi_{i}\right)=x_{i}^{T}\beta +k_{i}^{T}\underset{\overset{¯}{}}{t}heta,\left(3\right)$$

where K and $\underset{\overset{¯}{}}{t}heta$ are defined above. We make use of Pólya‐Gamma random variables to obtain the conditional distributions of the parameters of the above spatial occupancy model. The conditional distributions are very similar to those obtained for the SSO model although here we require the conditional posterior distribution of additional parameters ($\underset{\overset{¯}{}}{t}heta$ and τ). A directed acyclic graph of the spatial SSO model is displayed in Figure 2 below while in Table 2, we summarize the Gibbs algorithms for a spatial SSO model which employs Equation [Link] when using the PG method. The details regarding the algorithm can be found in the Supporting information (Appendix S4).

**Table 2:**
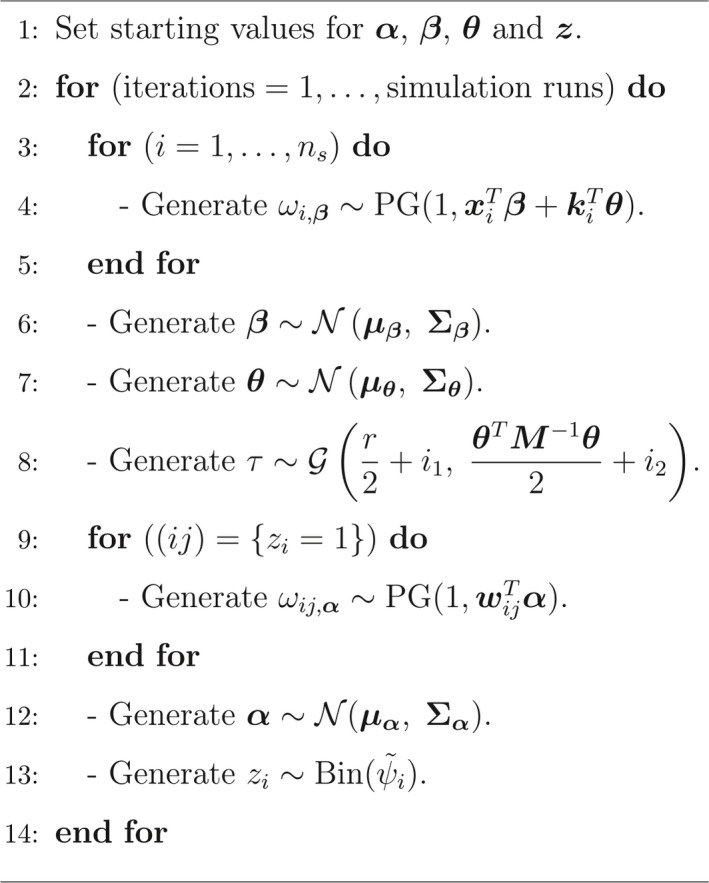
The Gibbs algorithm for undertaking a spatial SSO model (See the Supporting Information (Appendix S4) for the details pertaining to the parameter matrices of some of the conditional posterior distributions.)

### 2.3 Applications

To demonstrate our methods, we used detection/nondetection data extracted from the 2nd Southern African Bird Atlas Project (SABAP2) database to produce a species distribution map of the Cape weaver (*Ploceus capensis*) and helmeted guineafowl (*Numida meleagris*) for South Africa. SABAP2 divides Southern Africa into a continuous grid of 5′ × 5′ and relies on citizen scientists to collect checklists of bird species for each grid cell. Birders are requested to spend at least 2 hr on each checklist in which they undertake *intense birding* and record all species they observe and the order in which they are observed. For this analysis, we aggregated the data to quarter‐degree grid cells. We used data that span South Africa and contained a minimum of three and a maximum of fifty surveys during 2016 (January–December) in the analysis. Covariate information at unsurveyed locations was included in the analyses to obtain occupancy estimates that span South Africa. All covariates were centered and standardized.

In our analysis, we fitted a nonspatial and spatial SSO model with one detection covariate and two occupancy covariates. The detection covariate used was the number of species observed by the birder (denoted as *nspp*), while the occupancy covariates were functions of seven climate variables. It is assumed that the more species the birder observes while birding, the likely they are to observe the particular species being analyzed such that a positive detection regression effect is expected. The aim of the analysis is not to obtain *the best* occupancy model for the particular data sets but rather to highlight the use of the developed Gibbs sampling algorithm for fitting RSR occupancy models to the data using different software programs and different sampling methods. We specifically consider the Gibbs sampling algorithm by Johnson et al. (2013) (probit link functions), our Gibbs algorithm (logit link functions), *JAGS*, and *Stan* (which uses a *no‐U‐turn* Hamiltonian Monte Carlo sampler (Hoffman & Gelman, 2014) to sample from the parameters of a posterior distribution).

The climate variables (Figure S5 in the Supporting Information Appendix S6) all form part of a data set used by Huntley et al. (2006) to model bird distributions in Southern Africa. The variables included two measures of annual temperature that related to thermal sums above 0 and 5 degree centigrade; two measures related to the mean temperature of the coldest and warmest month, respectively; the ratio of potential to realized evapotranspiration as well as two measures that relates to the intensity of the dry and wet season, respectively. The climate variables are highly correlated with two of the variables having variance inflation factors in excess of 3,000 (Tables S2 and S3 in the Supporting Information Appendix S5). Because of this fact, it was decided to extract two principal components from the design matrix that consisted of the centered and standardized climate variables. These principal components explain 90% of the variation in the design matrix (Table S4 in the Supporting Information Appendix S5) and can tentatively be interpreted as a temperature related factor and a climate intensity factor, respectively.

We follow Hughes and Haran (2013) and retain 10% of the eigenvalues ($\lambda_{i}\;i\;=\;1,\ldots ,n$) of ω. In a similar context, Johnson et al. (2013) suggest selecting a RSR model with $\lambda_{i}\;\geq \;0.5$ which suggests including at most 237 eigenvectors into the spatial portion of the model. Experimentation with different values of r between 150 and 230 demonstrated no significant difference to the results we report here.

The following prior distributions were used for the parameters of the spatial SSO model: $\alpha ∼N(0,1000I_{2})$, $\beta ∼N(0,1000I_{3})$, and τ∼G(0.5,0.005). The prior specification for τ places more weight on large values of τ indicating that very little prior weight is placed on the spatial random effects of the model. Broms (2013) performed a simulation study and found that the RSR model results are not sensitive to the prior specification of τ and thus we have not done any analysis to test the sensitivity of our results to the prior specification of τ. All MCMC sampling was undertaken using the R packages, *stocc*,* jagsUI* (Kellner, 2014) in combination with *JAGS* 4.2.0 (Plummer, 2003), *rstan* in combination with *Stan* 2.17.3 as well as the authors’ code.^1^ All calculations were performed on a Windows 10 Pro desktop computer which had an Intel(R) Core(TM) i7‐6900 processor with 64 GB of RAM. One chain of 70,000 iterations was run. The first 20,000 samples were discarded as burn‐in samples, while the remaining samples were retained. Experimentation and an examination of the Geweke convergence diagnostic statistics (Geweke, 1992) and trace plots obtained by running three parallel chains using *Rcppocc* displayed that the MCMC chains converged using these numbers of iterations. The posterior samples were not thinned (Link & Eaton, 2012).

## 3 RESULTS

**Figure A1:**
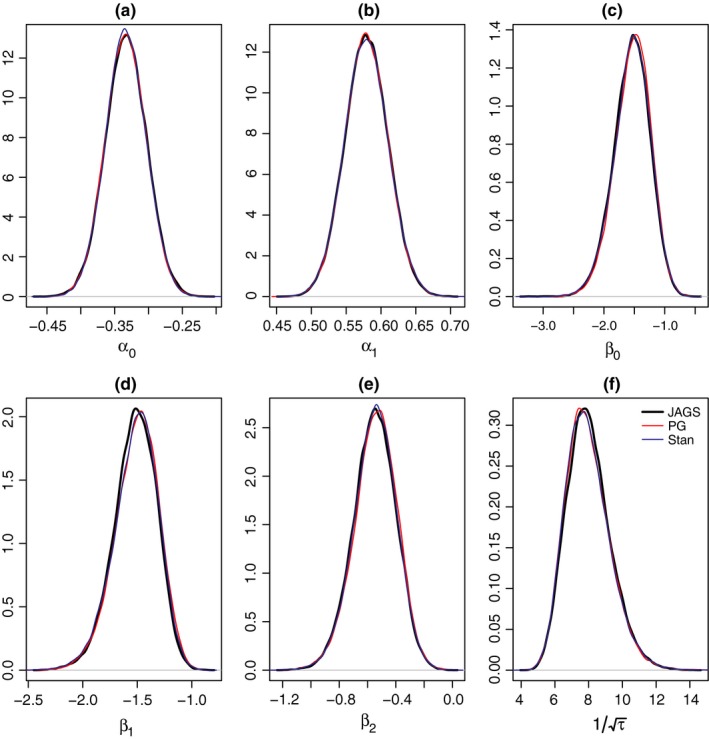
Posterior distributions of the parameters of the Bayesian spatial occupancy model using *JAGS*,* Stan*, and the Pólya‐Gamma formulation for the Cape weaver data set [(a) = $\alpha_{0}$, (b) = $\alpha_{1}$, (c) = $\beta_{0}$, (d) = $\beta_{1}$, (e) = $\beta_{2}$, (f) = $\frac{1}{\sqrt{\tau }}$]

**Figure A2:**
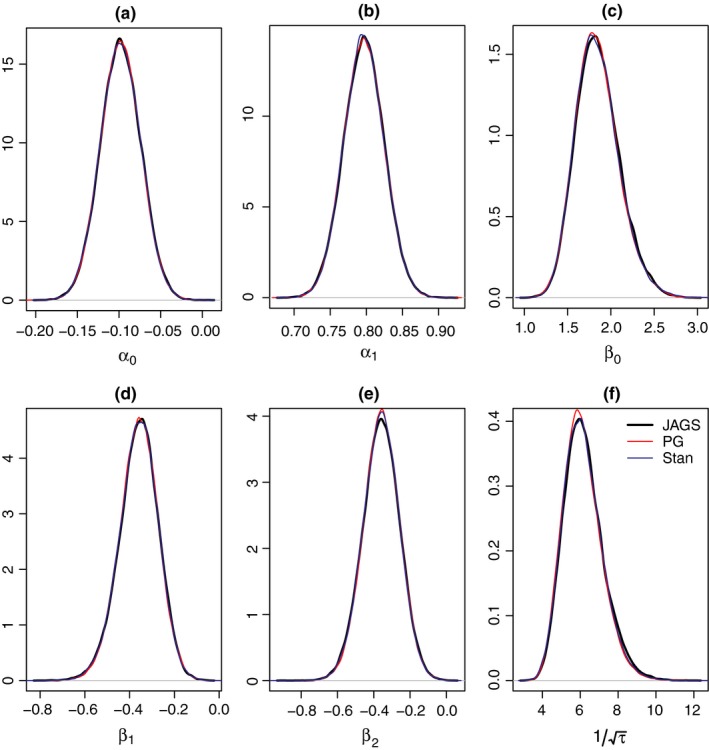
Posterior distributions of the parameters of the Bayesian spatial occupancy model using *JAGS*,* Stan*, and the Pólya‐Gamma formulation for the helmeted guineafowl data set [(a) = $\alpha_{0}$, (b) = $\alpha_{1}$, (c) = $\beta_{0}$, (d) = $\beta_{1}$, (e) = $\beta_{2}$, (f) = $\frac{1}{\sqrt{\tau }}$]

**Figure A3:**
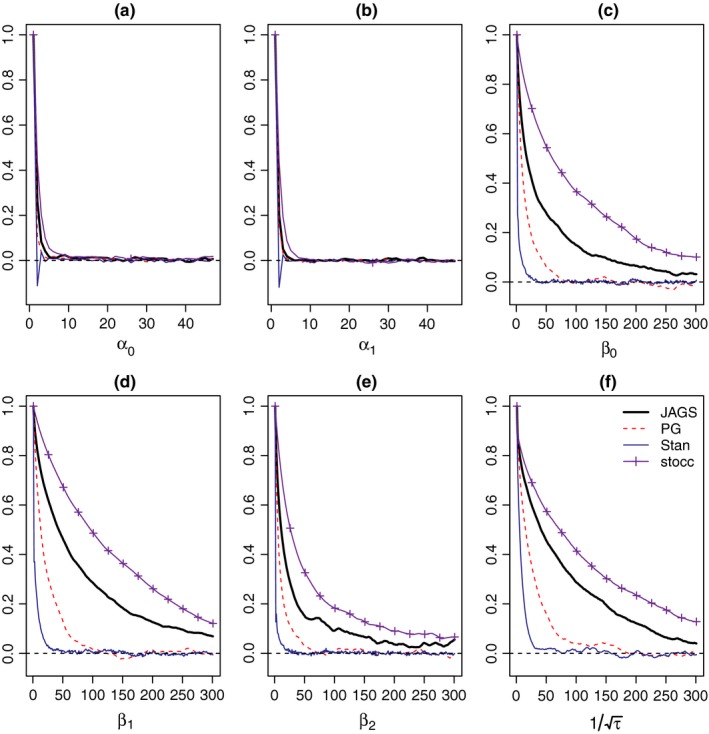
Estimated lagged sample autocorrelations of the posterior samples of the parameters of the Bayesian spatial occupancy model using *JAGS*, the Pólya‐Gamma formulation, *Stan* and *stocc* for the Cape weaver data set [(a) = $\alpha_{0}$, (b) = $\alpha_{1}$, (c) = $\beta_{0}$, (d) = $\beta_{1}$, (e) = $\beta_{2}$, (f) = $\frac{1}{\sqrt{\tau }}$]

**Figure A4:**
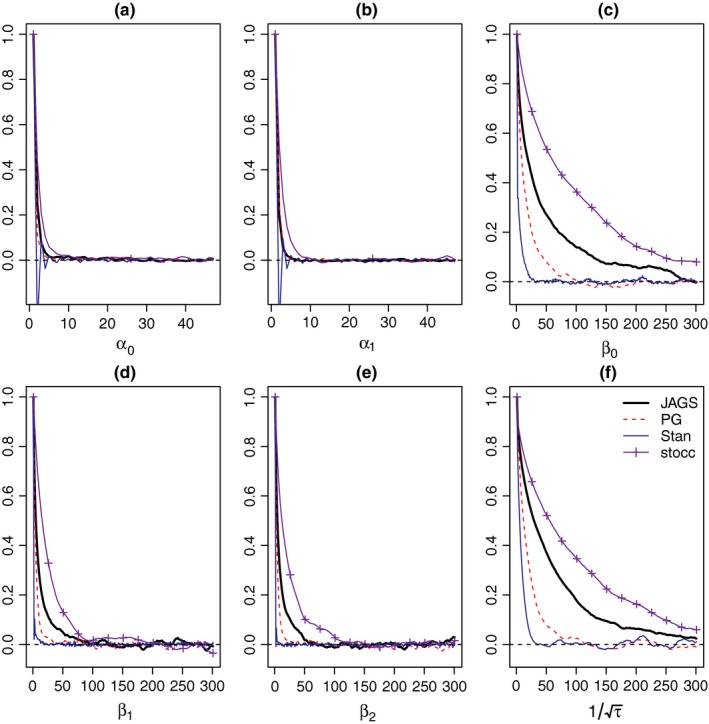
Estimated lagged sample autocorrelations of the posterior samples of the parameters of the Bayesian spatial occupancy model using *JAGS*, the Pólya‐Gamma formulation, *Stan* and *stocc* for the helmeted guineafowl data set [(a) = $\alpha_{0}$, (b) = $\alpha_{1}$, (c) = $\beta_{0}$, (d) = $\beta_{1}$, (e) = $\beta_{2}$, (f) = $\frac{1}{\sqrt{\tau }}$]

From the analysis of both data sets, we observe that the Gibbs algorithm developed for the spatial occupancy model produces identical posterior distributions to those obtained when using *JAGS* and *Stan* (Figures A1 and A2 in Appendix 1). In both data sets, the posterior samples of the detection regression effects exhibit good mixing where the lagged sample autocorrelations of the posterior samples approach zero within 5 lags. The posterior samples of the occupancy regression effects as well as the precision of the spatial random effect (τ) exhibit slower mixing when using *stocc*,* JAGS*, and *Rcppocc* (denoted as “PG” in Figures A3 and A4 in Appendix 2), while *Stan* produced a posterior chain that mixed well. We observe that *stocc* produced posterior samples for α, β, and τ that had the largest levels of autocorrelation among all of the methods considered (when fitting the RSR model).

Table 3 tabulates the run times (in minutes) and effective sampling rate (ESR^2^  = the effective sample size per unit run time) of ***α***,***β***, and *τ* for each of the sampling algorithms used to analyze the two data sets. For completeness sake, we also include the statistics related to the ICAR model when using *stocc*. We observe that *stocc* and *Rcppocc* had faster run times than *JAGS* and *Stan*. *Rcppocc* had the fastest running times and completed the 70,000 MCMC iterations approximately 12 times faster than *JAGS* and between 7 and 10 times faster than *Stan*. *Rcppocc* has the largest ESR of all of the algorithms considered and produced ESR values which ranged between 1.5 and 6 times larger than those obtained by *Stan*; 3–11 times larger than those obtained by *stocc* and 11–60 times larger than those obtained by *JAGS*. The ICAR models took approximately 8 times longer to run than the RSR model when using *stocc* and resulted in significantly larger levels of autocorrelation within the α, β, and τ chains.

**Table 3:** Posterior run times for the Bayesian spatial occupancy models as well as the ESR (per minute) for α, β, and τ

Species	Method	Time (min)	$\alpha_{0}$	$\alpha_{1}$	$\beta_{0}$	$\beta_{1}$	$\beta_{2}$	τ
Cape weaver	*stocc* (RSR)	27.00	552.71	725.59	12.03	7.83	22.37	11.35
	*stocc* (ICAR)	136.76	104.41	142.57	0.25	0.10	0.15	0.14
	*JAGS*	243.55	102.62	131.42	2.69	1.71	5.09	1.97
	*Stan*	187.06	275.75	331.44	53.48	34.42	83.74	19.35
	*Rcppocc*	19.88	1682.15	1804.42	85.32	53.40	141.82	116.19
Helmeted Guinea fowl	*stocc* (RSR)	27.08	619.72	563.24	11.63	43.61	48.34	15.61
	*stocc* (ICAR)	165.23	65.92	97.15	0.04	0.16	0.09	0.05
	*JAGS*	254.49	97.86	125.35	3.08	10.84	12.06	2.66
	*Stan*	150.55	595.77	617.84	49.21	186.23	286.92	24.44
	*Rcppocc*	19.9	1888.11	1493.07	86.16	314.04	364.47	106.06

The posterior summaries for some of the parameters of the nonspatial and spatial model are displayed in Table 4. The fixed regression effects of all of the parameters (for both data sets) were statistically different from zero since none of the 95% highest density credibility interval of the parameters contained zero. In all cases, the regression effect for *nspp* was positive (as expected), while the regression effects of the occupancy effects were negative. The detection regression effects for both model types (for the respective species) were identical. The regression effects for the occupancy process for the Cape weaver were significantly different for the two model types, while the same regression effects for the helmeted guineafowl were identical for both model types (except for the intercept). The posterior distribution of the spatial standard deviation parameter ($\sigma \;=\;1/\sqrt{\tau }$ indicates that the spatial process does significantly contribute to the variability of the occupancy process across South Africa. The 95% posterior highest density credibility interval for σ is [5.56, 10.59] and [4.26, 8.42] for the Cape weaver and the helmeted guineafowl data sets, respectively.

**Table 4:** Posterior summaries of the parameters of the Bayesian nonspatial and spatial occupancy models (posterior mean, Monte Carlo standard error, standard deviation, 2.5% and 97.5% quantiles)

Type	Species	Parameter	Mean	MCSE	*SD*	2.5%	97.5%
Nonspatial	Cape weaver	$\alpha_{0}$	−0.32	0.0002	0.03	−0.38	−0.26
		$\alpha_{1}$	0.56	0.0002	0.03	0.49	0.62
		$\beta_{0}$	−0.49	0.0006	0.10	−0.68	−0.30
		$\beta_{1}$	−0.71	0.0005	0.06	−0.84	−0.59
		$\beta_{2}$	−0.24	0.0004	0.06	−0.36	−0.12
	Helmeted guineafowl	$\alpha_{0}$	−0.10	0.0001	0.02	−0.15	−0.06
		$\alpha_{1}$	0.78	0.0002	0.03	0.72	0.84
		$\beta_{0}$	−0.35	0.0006	0.06	−0.48	−0.22
		$\beta_{1}$	−0.36	0.0005	0.09	−0.54	−0.20
		$\beta_{2}$	−0.39	0.0008	0.08	−0.56	−0.24
Spatial	Cape weaver	$\alpha_{0}$	−0.33	0.0001	0.03	−0.39	−0.27
		$\alpha_{1}$	0.58	0.0001	0.03	0.52	0.64
		$\beta_{0}$	−1.54	0.0030	0.30	−2.17	−1.00
		$\beta_{1}$	−1.51	0.0027	0.20	−1.96	−1.16
		$\beta_{2}$	−0.55	0.0012	0.15	−0.86	−0.27
		τ	0.02	0.0001	0.01	0.01	0.03
	Helmeted guineafowl	$\alpha_{0}$	−0.10	0.0001	0.02	−0.15	−0.05
		$\alpha_{1}$	0.80	0.0001	0.03	0.74	0.85
		$\beta_{0}$	1.85	0.0029	0.25	1.40	2.40
		$\beta_{1}$	−0.36	0.0005	0.09	−0.54	−0.20
		$\beta_{2}$	−0.36	0.0004	0.10	−0.56	−0.17
		τ	0.03	0.0002	0.01	0.01	0.05

**Figure 3:**
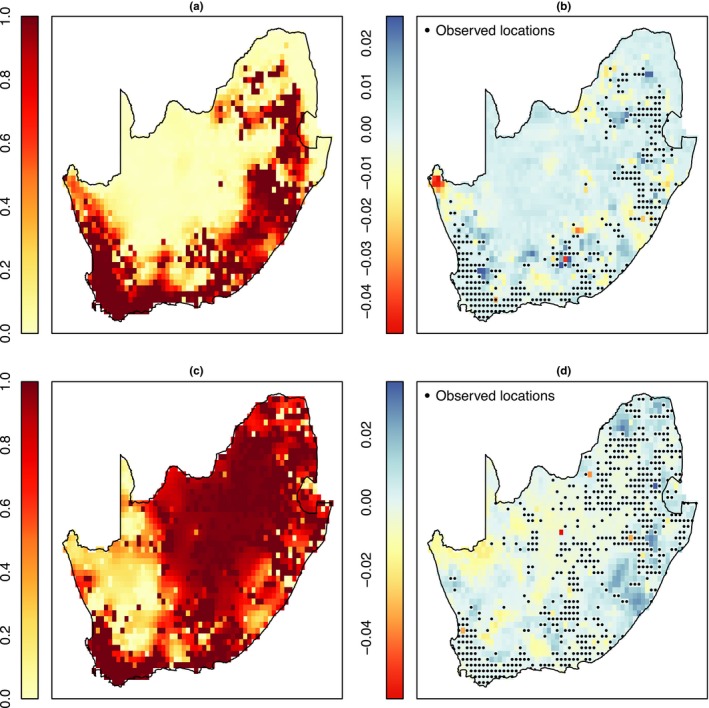
Estimated occupancy probability for the Cape weaver and helmeted guineafowl estimated using *Rcppocc* (a and c). The difference between the estimated occupancy probabilities obtained when using *Rcppocc* and *stocc* for the Cape weaver and helmeted guineafowl, respectively (b and d). The grid cells where the species have been detected at least once are displayed in (b) and (d)

Figure 3a,c displays the estimated occupancy probabilities ($\text{Pr}\;(z_{i}\;=\;1|.)$) across South Africa estimated using *Rcppocc* for the Cape weaver and helmeted guineafowl data set, respectively. The figures illustrate that there is a high probability that the Cape weaver occupies coastal regions throughout South Africa and low occupancy probability (close to zero) in the interior areas of South Africa. In contrast, the helmeted guineafowl has very high occupancy probabilities in most regions of South Africa except for the North West regions of South Africa. Figure 3b,d displays the difference between the estimated occupancy probabilities obtained when using *Rcppocc* and *stocc*, respectively (“*Rcppocc‐stocc*”). The figures illustrate that we obtain similar estimates of the mean occupancy probabilities when using either estimation method with small discrepancies at the majority of the grid cells across South Africa.

## 4 DISCUSSION AND CONCLUSIONS

Through several studies, Bayesian methods have been developed to undertake occupancy models. They, however, either use probit link functions to model the detection and occupancy processes of the model; use general Bayesian analysis software such as *JAGS*,* WinBUGS*,* OpenBUGS*, or *Stan* to undertake their analysis or make use of the Metropolis–Hastings algorithm to sample from the parameters of the model. We develop a Gibbs sampling algorithm to obtain posterior samples of the parameters of a restricted spatial regression (RSR) occupancy model and demonstrate that the method has a larger expected sampling rate (ESR) and faster run times when compared to previous Bayesian methods used in the literature to date.

Similar to Broms, Johnson, Altwegg, and Conquest (2014) and Johnson et al. (2013), we show that the ICAR model produced posterior samples with significantly larger autocorrelations than the RSR model when using *stocc*. As an example, the autocorrelations of the occupancy regression effects as well as the spatial precision parameter (of both data sets) had autocorrelations in excess of 0.7 at lag 500 indicating that the posterior chain of the model mixed poorly for those parameters of the ICAR model. Additionally, the run times of the ICAR model were approximately 5 times longer than the run times of the RSR model and thus we do not recommend its use when fitting a spatial occupancy model.

Based on the two data sets, we observed that the new algorithm not only ran faster (approximately 35%) than the Gibbs sampler implemented in *stocc*, it also generated expected sample size (ESS) statistics between 2 and 6 times larger than those obtained using *stocc*. The main reason for the time difference is that *stocc* has been coded using R, while *Rcppocc* uses Rcpp and RcppArmadillo to undertake all matrix computations. *Stan* uses compiled C++ code to implement the *no‐U‐turn* Hamiltonian Monte Carlo algorithm and generated ESS statistics between 2 and 7 times larger than those obtained using *Rcppocc*. In many applications, *Stan* has been shown to be much faster than *JAGS* although at present *Stan* has run times that are approximately 7–10 times slower than *Rcppocc* when fitting spatial occupancy models. The opportunity thus exists to develop suitable *Stan* (or NIMBLE) code that can fit spatial occupancy models in a shorter period of time.

## CONFLICT OF INTEREST

The authors have no conflict of interests to declare.

## AUTHOR CONTRIBUTION

Below, Allan Ernest Clark is denoted as “AEC”, while Res Altwegg is denoted as “RA”. AEC and RA conceived and designed the paper. AEC analyzed the data. AEC wrote and, AEC and RA reviewed the paper. AEC designed and coded the software used in the analysis. AEC wrote computer code used to perform all analysis.

## Notes

1 An R package has been developed to fit these models using MCMC. All code can be obtained from https://github.com/AllanClark/Rcppocc. Appendix S7 in the Supporting Information includes a worked example explaining how to run RSR models using *stocc*,* Stan*, and *Rcppocc*.

2 ESR = the effective sample size per unit run time. The effective sample size for the ith parameter in the model is defined as $ESS_{i}\;=\;M/1\;+\;2\sum_{j\;=\;1}^{k}\rho_{i}\left(j\right),$ where M is the number of retained samples, and $\rho_{i}\left(j\right)$ is the $j^{\mathrm{th}}$ lagged autocorrelation of parameter i (Holmes & Held, 2006). We use the *coda* package (Plummer, Best, Cowles, & Vines, 2006) to estimate ${\text{ESS}}_{i}$.
