# Supplementary material for: Efficient Bayesian analysis of occupancy models with logit link functions
Source: Ecol Evol. 2019 Feb 5;9(2):756–68. doi: 10.1002/ece3.4850 (PMC6362608; doi:10.1002/ece3.4850)
Supplement: Supplementary file 1 [file ECE3-9-756-s001.zip › Supp.pdf]

1 Supporting information - Appendices

The following sections contain Appendices to the manuscript.

1.1 Gibbs algorithms to undertake logistic regression

A number of Gibbs sampling algorithms have been developed to obtain samples from the posterior distribution of the parameters of a **logistic regression model**. Holmes and Held 2006 represent the logistic distribution as a normal-mixture that utilizes a Kolmogorov-Smirnov distribution (Andrews and Mallows 1974) as the mixing distribution while Frühwirth-Schnatter and Frühwirth 2007 and Frühwirth-Schnatter and Frühwirth 2010 both utilize the random-utility formulation of Mcfadden 1974 to develop their RUM (random utility method) and dRUM (difference of random utility method) algorithms. Both methods use *various approximations* as part of their algorithms. With the use of Pólya-Gamma random variables[1], Polson et al. 2013 developed a simple sampling scheme that generates samples from the correct posterior distribution without the aid of additional hierarchical levels or approximations. They evaluated the mixing properties of the above methods and found that **their method** performed best although Frühwirth-Schnatter and Frühwirth 2010 appeared to be a good alternative algorithm. Below we briefly discuss both of these formulations and explain how they could be used to obtain posterior samples for the parameters of the single-season occupancy (SSO) model.

Following Frühwirth-Schnatter and Frühwirth 2010, the logistic regression model can be formulated using unobserved latent random variables z_i^{u*}, for $i = 1, \ldots, n_s$ where n_s is

[1]"A random variable X has a Pólya-Gamma distribution with parameters $b > 0$ and $c \in \Re$, denoted $X \sim \text{PG}(b, c)$, if

$$X \stackrel{D}{=} \frac{1}{2\pi^2} \sum_{k=1}^{\infty} \frac{g_k}{(k-0.5)^2 + c^2/(4\pi^2)},$$

where $g_k \sim \mathcal{G}(b, 1)$ are independent gamma random variables, and where $\stackrel{D}{=}$ indicates equality in distribution" (Polson et al. 2013).

the number of surveyed sites. These random variables are modelled using explanatory variables with design matrix $\boldsymbol{X}$, while the error of the relationship is modelled using a Logistic distribution[2] . We thus have

$$z_i^{u*} = \boldsymbol{x}_i^T \boldsymbol{\beta} + \epsilon_i, \ \epsilon_i \sim \text{Logistic}(0,1) \text{ with } y_i = I_{\{z_i^{u*}>0\}}, \ \text{for all } i = 1, \ldots, n_s.$$

The novelty of the method lies in the approximation of $p(\epsilon)$ using a linear Gaussian mixture distribution with **known weights**, w_r^* and variances, s_r^2 for $r = 1, 2, 3$. Since the Logistic distribution is symmetric about 0, the means of the Gaussian mixture distribution are assumed to be 0 as well. The logistic model is now reformulated using the following hierarchical framework,

$$z_i^{u*} = \boldsymbol{x}_i^T \boldsymbol{\beta} + \epsilon_i, \tag{1.1}$$

$$\epsilon_i | r_i \sim \mathcal{N}(0, \omega_i) \text{ with } \omega_i = s_{r_i}^2 \text{ and} \tag{1.2}$$

$$r_i \sim \text{Multinomial}(w_1, w_2, w_3), \ \text{for all } i = 1, \ldots, n_s. \tag{1.3}$$

The use of the linear Gaussian approximation allows one to perform weighted linear regression to obtain the posterior distribution of the regression coefficients conditional on knowing the $\boldsymbol{z}^{u*}$ vector as well as $\boldsymbol{w} = (w_1, w_2, w_3)^T$ and $\boldsymbol{\omega} = (\omega_1, \omega_2, \omega_3)^T$. The conditional posterior distributions of z_i^{u*} and r_i are easily obtained using equations (1.1-1.3) in the framework above.

Polson et al. 2013 show that posterior samples of $\boldsymbol{\beta}$ can be obtained by sampling from the following conditional distributions, in turn,

$$\omega_i | \boldsymbol{\beta} \sim \text{PG}(1, \boldsymbol{x}_i^T \boldsymbol{\beta}), \ \text{for all } i = 1, \ldots, n_s$$

$$\boldsymbol{\beta} | \boldsymbol{y}, \boldsymbol{\omega} \sim \mathcal{N}(\boldsymbol{\mu}, \ \boldsymbol{v})$$

where the PG notation represents a Pólya-Gamma random variable, $\boldsymbol{\mu}$ and $\boldsymbol{v}$ are the mean vector and covariance matrix of a multivariate Gaussian distribution.

[2]The probability density function of a Logistic distribution with parameters $a \in \Re$ and $b > 0$ is $f(x) = \dfrac{e^{-(x-a)/b}}{b(1+e^{-(x-a)/b})^2}$ (Balakrishnan 1992).

Below we briefly investigate how both of these methods could be used to sample from the posterior distribution of the parameters of a SSO model, with the aim of deciding which of the two formulations (Appendix 1.2 and Appendix 1.3) should be used to develop a Gibbs sampling algorithm for spatial occupancy models.

1.1.1 A Gibbs algorithm for the SSO model

Since an occupancy model has regression effects in both the occupancy and detection process, two sets of additional random variables are introduced into the sampling algorithm. The dRUM method (Algorithm 1 in Table S1) thus require posterior samples for $\boldsymbol{z}^{u*}$, $\boldsymbol{r}$, $\boldsymbol{y}^{u*}$ and $\boldsymbol{r}^{*}$, where the first two relate to the occupancy regression effects and the last two relates to the detection regression effects. Algorithm 2 (Table S1) can be used to generate samples from two sets of Pólya-Gamma random variables, $\boldsymbol{\omega_\alpha}$ and $\boldsymbol{\omega_\beta}$, in order to obtain posterior samples for the regression effects of an SSO model.

Table S1. Two Gibbs algorithms for a SSO model. (See Appendix 1.2 and 1.3 for the details pertaining to the parameter matrices of the conditional posterior distributions.)

Algorithm 1. (DRUM)

Set starting values for $\boldsymbol{\beta}$ and $\boldsymbol{z}$.
for (iterations $= 1, \ldots,$ simulation runs) **do**
  for ($i = 1, \ldots, n_s$) **do**
    - Generate $z_i^{u*} \sim [z_i^{u*} | \boldsymbol{z}, \boldsymbol{\beta}]$.
    - Generate $r_i \sim [r_i | z_i^{u*}, \boldsymbol{\beta}]$.
  end for
  - Generate $\boldsymbol{\beta} \sim \mathcal{N}\left(\boldsymbol{\mu_\beta},\ \boldsymbol{\Sigma_\beta}\right)$.
  for ($(ij) = \{z_i = 1\}$) **do**
    - Generate $y_{ij}^{u*} \sim [y_{ij}^{u*} | \boldsymbol{y}, \boldsymbol{\alpha}]$.
    - Generate $r_{ij}^{*} \sim [r_{ij}^{*} | \boldsymbol{y}, \boldsymbol{y}^{u,*}]$.
  end for
  - Generate $\boldsymbol{\alpha} \sim \mathcal{N}(\boldsymbol{\mu_\alpha},\ \boldsymbol{\Sigma_\alpha})$.
  - Generate $z_i \sim [z_i | \boldsymbol{\alpha}, \boldsymbol{\beta}, \boldsymbol{y}]$.
end for

Algorithm 2. (PG)

Set starting values for $\boldsymbol{\beta}$ and $\boldsymbol{z}$.
for (iterations $= 1, \ldots,$ simulation runs) **do**
  for ($i = 1, \ldots, n_s$) **do**
    - Generate $\omega_{i,\beta} \sim \mathrm{PG}(1, \boldsymbol{x}_i^T \boldsymbol{\beta})$.
  end for
  - Generate $\boldsymbol{\beta} \sim \mathcal{N}\left(\boldsymbol{\mu_\beta},\ \boldsymbol{\Sigma_\beta}\right)$.
  for ($(ij) = \{z_i = 1\}$) **do**
    - Generate $\omega_{ij,\alpha} \sim \mathrm{PG}(1, \boldsymbol{w}_{ij}^T \boldsymbol{\alpha})$.
  end for
  - Generate $\boldsymbol{\alpha} \sim \mathcal{N}(\boldsymbol{\mu_\alpha},\ \boldsymbol{\Sigma_\alpha})$.
  - Generate $z_i \sim [z_i | \boldsymbol{\alpha}, \boldsymbol{\beta}, \boldsymbol{y}]$.
end for

Table S1 displays a summary of the sampling scheme required to sample from the posterior distribution of the parameters associated with the SSO model for the two Gibbs sampling algorithms considered in the paper. It should be immediately apparent that Algorithm 1 requires **twice** as many additional parameters to sample from than Algorithm 2. Algorithm 2 however requires samples from a Pólya-Gamma distribution which requires the use of an acceptance-rejection algorithm (Robert and Casella 1999). Both algorithms have been coded using the R programming language (R Core Team 2014) in conjunction with *RcppArmadillo* (Sanderson and Curtin 2016) and *Rcpp* (Eddelbuettel and Francois 2011). The Pólya-Gamma random variables were generated by amending the Rcpp code in the R package *Binomlogit* (Windle et al. 2013). The exact details related to the algorithms can be found in Appendix 1.2 and Appendix 1.3.

1.1.2 Simulation Study

As noted above, Polson et al. 2013 found that the use of Pólya-Gamma random variables lead to MCMC chains that **mixed faster** than alternate algorithms when undertaking **logistic regression**. Below we investigate whether or not this observation holds when applying Algorithm 2 to simulated detection/non-detection data. We utilize the effective sample size (ESS) and the effective sampling rate (ESR) (effective sample size per second of run-time) to compare the efficiency of MCMC chains produced using *JAGS*, Algorithm 1, Algorithm 2 and *Stan*. The effective sample size for the ith parameter in the model is defined as

$$ESS_i = \frac{M}{1 + 2\sum_{j=1}^{k} \rho_i(j)},$$

where M is the number of post-burn-in samples, and $\rho_i(j)$ is the jth lagged autocorrelation of parameter i (Holmes and Held 2006). We use the *coda* package (Plummer et al. 2006) to estimate ESS_i and use boxplots of the median (*across all regression effects*) effective sample size and median effective sampling rate to draw conclusions regarding the efficiency of the four sampling methods.

The simulation settings discussed below are very similar to that of Clark et al. 2016. We

consider the following simulation settings. The number of sites (n_s) are set to 50 and 100 while the number of surveys to each site (J) are set to 3 and 5 respectively. The following combinations of the regression coefficients are used: 1. $\boldsymbol{\alpha} = [0,\ 1.75]^T$, $\boldsymbol{\beta} = [-1.85,\ 2.5]^T$; 2. $\boldsymbol{\alpha} = [0,\ 1.75]^T$, $\boldsymbol{\beta} = [-0.1,\ 2.5]^T$; 3. $\boldsymbol{\alpha} = [1.35,\ 1.75]^T$ $\boldsymbol{\beta} = [-1.85,\ 2.5]^T$ and 4. $\boldsymbol{\alpha} = [1.35,\ 1.75]^T$, $\boldsymbol{\beta} = [-0.1,\ 2.5]^T$. These parameter values ensure an approximate average detection and occupancy probability among the sites of (0.5, 0.3), (0.5, 0.5), (0.7, 0.3) and (0.7, 0.5) respectively. In order to assess how well these algorithms perform for larger sample sizes we also consider the following cases, $n_s = 500$ with $J = 5$ and $J = 10$ using the first two parameter settings described above.

"The occupancy regression covariate was obtained by standardizing a Uniform $(-2, 2)$ random variable while the detection covariate was obtained by standardizing a Uniform$(-5, 5)$ random variable. Each of these variables were transformed to have a zero mean and a standard deviation of one. The following parameter vectors were used to specify the prior distribution of the parameters: $\boldsymbol{\mu}_{\boldsymbol{i}}^0 = [0, 0]^T$, $\boldsymbol{\Sigma}_{\boldsymbol{i}}^0 = \text{diag}[1000, 1000]$ for $\boldsymbol{i} = \boldsymbol{\alpha}, \boldsymbol{\beta}$." (Clark et al. 2016)

Each simulation setting was replicated 500 times. All calculations were undertaken using R 3.4.1 (R Core Team 2014). MCMC sampling was undertaken using the R packages *jagsUI* (Kellner 2014) and *rstan* in combination with *JAGS* 4.2.0 (Plummer 2003) and *Stan* 2.17.3 (Carpenter et al. 2017) while Algorithm 1 and Algorithm 2 were performed using the authors' code. A copy of the R package created to undertake the posterior sampling from the SSO model can be found at `https://github.com/AllanClark/Rcppocc`. Note that the **default settings** for the *Stan* function, *sampling*, were used in order to undertake sampling when using *Stan*. 20 000 posterior samples was obtained for each MCMC simulation. The first 10 000 samples were discarded as burn-in samples while the remaining samples were retained. Prior experimentation using the MCMC algorithm indicated that the Markov chains would converge to the stationary distributions using this number of posterior samples. The posterior samples were not thinned (Link and Eaton 2012).

1.1.3 Results: Simulation Study

Based on the simulation study, we conclude that Algorithm 2 had the fastest **run-times** across all scenarios considered. *JAGS* and Stan had similar run-times for $n_s \leq 100$ with median run-times being between 6-12 slower than Algorithm 2. The *JAGS* algorithm does not scale well and took $40 - 70$ times longer to complete than Algorithm 2 when $n_s = 500$ and comparisons are made using the median run-times across of all regression effects. We found that Algorithm 1 performed well relative to Algorithm 2 except for $n_s = 500$ and in general we can conclude that for small sample sizes the run-times for *JAGS* and *Stan* are similar although *Stan* outperforms *JAGS* for large sample sizes (Figure S1). A similar result was found by Monnahan et al. 2017 when investigating various population ecology models.

We found that the median ESS obtained when using *Stan* is significantly larger than those obtained when using the other three algorithms. *JAGS* and Algorithm 1 produced similar ESS's while Algorithm 2 produced larger ESS's than *JAGS* and Algorithm 1 in general (Figures S2 and S3).

The median ESR obtained when using Algorithm 2 is **generally much larger** than the median ESR obtained when using the other three algorithms and occurs largely due to the faster run-times of Algorithm 2 (Figures S2 and S3). For $n_s \leq 100$, the ESR for Algorithm 2 is $3 - 12$ larger than those obtained when using Stan; $3 - 5$ larger than those obtained when using Algorithm 1 and $10 - 120$ larger than those obtained when using JAGS (*when one does not use the median regression effects to combine the results)*. When $n_s = 500$, Algorithm 2 performs significantly better than all other algorithms with the ESR being $3 - 10$ larger than those obtained when using Stan, $15 - 60$ larger than those obtained when using Algorithm 1 and $30 - 450$ larger than those obtained when using *JAGS*.

The above observations are broadly consistent across all scenarios considered. Regarding large data sizes; we can conclude that this negatively effects ESR although Algorithm 2 clearly outperforms the other three sampling algorithms. The poor performance of *JAGS* was expected and occurs since the software has been programmed to implement Bayesian

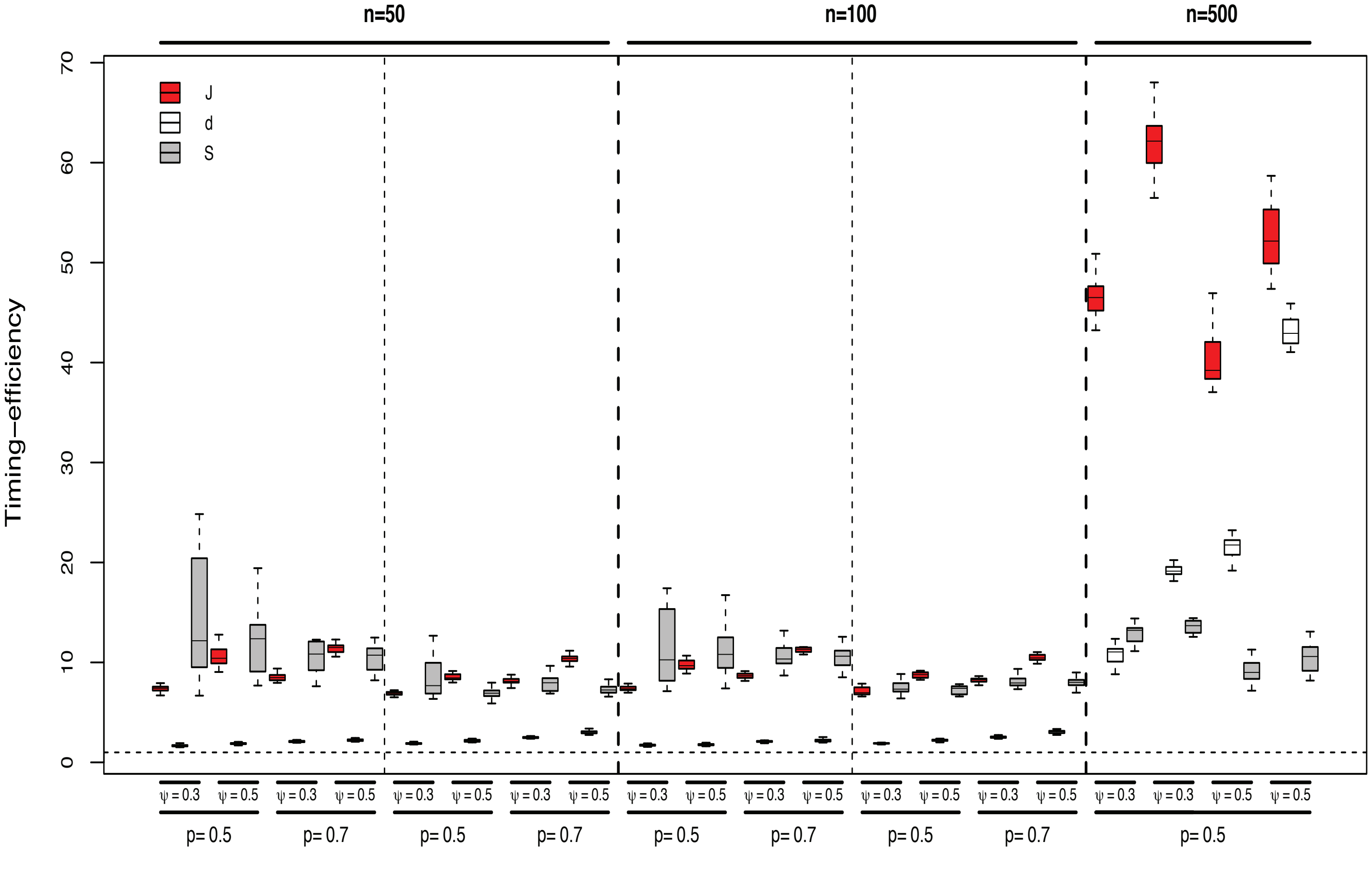

Figure S1. Trimmed Box plots of the relative efficiency (run-time of an algorithm in seconds divided by the run-time of Algorithm 2 in seconds) of *JAGS* (J), Algorithm 1 (d) and *Stan* (S) when generating 20 000 posterior samples from the posterior distribution of the SSO model for all simulation scenarios considered. The average occupancy probabilities and conditional detection probabilities are denoted as ψ and p. The horizontal dashed line represents a time-efficiency of 1.

analysis in many different situations. The software has not been written or tailored for speed and efficiency whereas Algorithms 1 and 2 were custom built to undertake the Bayesian analysis of SSO models using Rcpp. Recall also that Algorithm 1 requires the sampling of twice as many additional parameters than Algorithm 2. Algorithm 2 performed surprisingly well relative to *Stan*. *Stan* does not allow for sampling of discrete random variables and in an occupancy modelling context, we **marginalised** all true occupancy variables from the posterior distribution which results in much less parameters being

sampled.

Based on the **above** simulation results we propose that Pólya-Gamma random variables (instead of the dRUM formulation) be used to develop a Gibbs sampling algorithm for a spatial occupancy model (Appendix 1.4 below for such an algorithm). We have not extensively compared the two algorithms when fitting spatial occupancy models although believe that similar conclusions as was found above would be obtained (Figure S4).

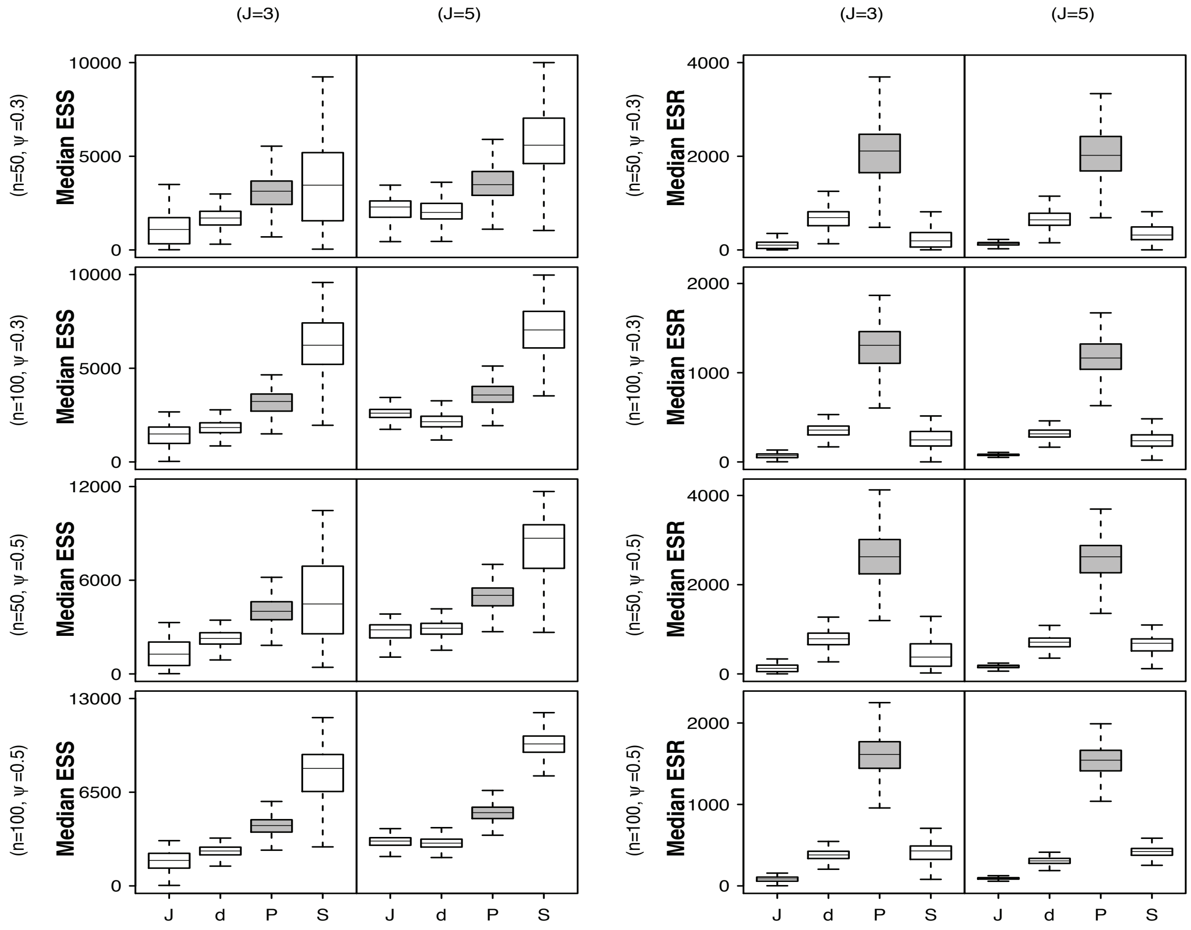

Figure S2. Trimmed Box plots of the median effective sample size (ESS) and median effective sampling rate (ESR) associated with MCMC chains produced using *JAGS* (J), Algorithm 1 (d), Algorithm 2 (P) and *Stan* (S) when fitting a nonspatial occupancy model. The number of sites are set to $n_s = 50$ and $n_s = 100$ while the number of surveys at each site are set to $K = 3$ and $K = 5$. The detection probability for these scenarios are approximately 0.5.

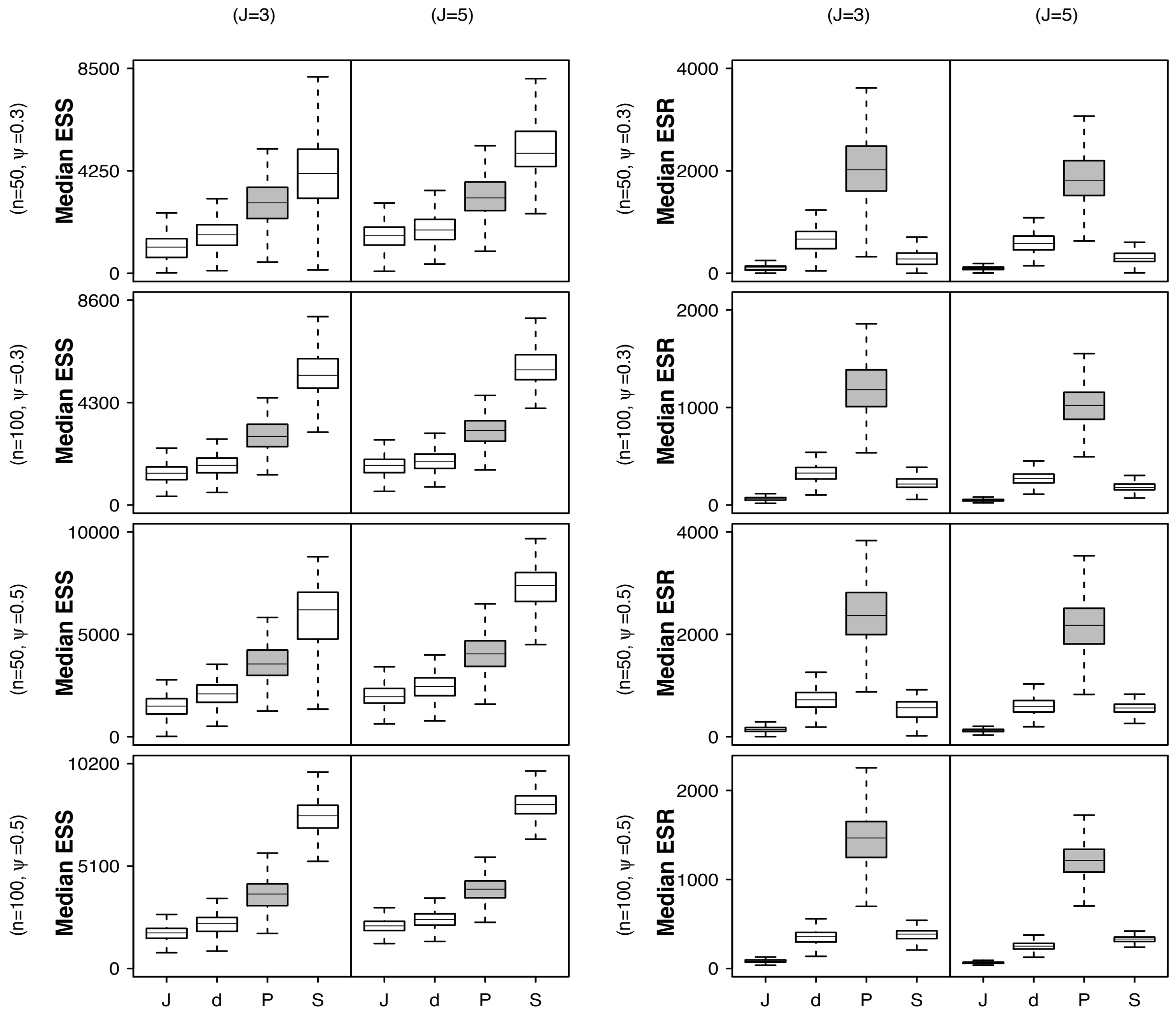

Figure S3. Trimmed Box plots of the median effective sample size (ESS) and median effective sampling rate (ESR) associated with MCMC chains produced using *JAGS* (J), Algorithm 1 (d), Algorithm 2 (P) and *Stan* (S). The number of sites are set to $n_s = 50$ and $n_s = 100$ while the number of surveys to each site are set to $K = 3$ and $K = 5$. The detection probability for these scenarios are approximately 0.7.

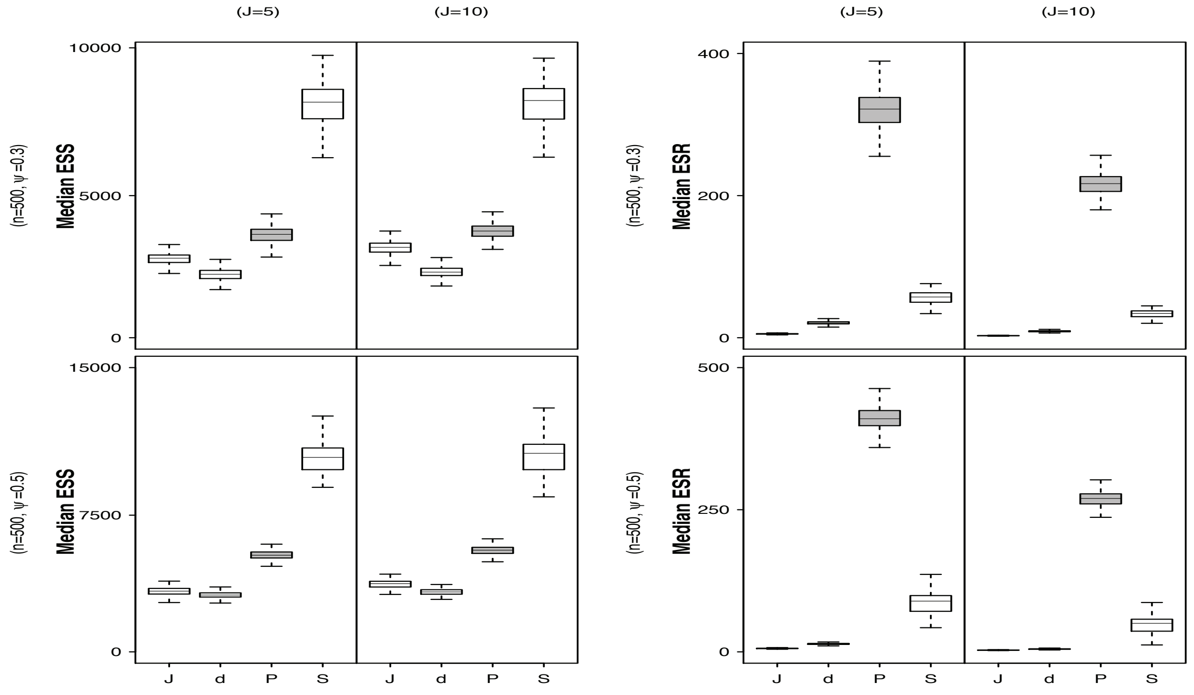

Figure S4. Box plots of the median effective sample size (ESS) and median effective sampling rate (ESR) associated with MCMC chains produced using *JAGS* (J), Algorithm 1 (d), Algorithm 2 (P) and *Stan* (S). The number of sites are set to $n_s = 500$ while the number of surveys to each site are set to $K = 5$ and $K = 10$. The detection probability for these scenarios are approximately 0.5.

1.2 Gibbs algorithm for the SSO model using dRUM.

Below we provide the full conditional distributions necessary to implement a Gibbs sampler for the SSO model when using the dRUM formulation. Here we assume that the prior distributions are $\boldsymbol{\alpha} \sim \mathcal{N}(\mathbf{0},\ \boldsymbol{\Sigma}^0_{\boldsymbol{\alpha}})$ and $\boldsymbol{\beta} \sim \mathcal{N}(\mathbf{0},\ \boldsymbol{\Sigma}^0_{\boldsymbol{\beta}})$. In addition, let $\boldsymbol{X}$ be a design matrix for the occupancy process with rows $\boldsymbol{x}_i^T$ and $\boldsymbol{W}$ be the design matrix for the detection process with rows $\boldsymbol{w}_{ij}^T$. If these prior distributions are adopted the Gibbs sampler proceeds as follows.

1. Set starting values for $\boldsymbol{\alpha}$, $\boldsymbol{\beta}$ and $\boldsymbol{z}$.

2. Cycle:

   2.1 Generate the latent utilities z_i^{u*}, $i = 1, \ldots, n_s$ conditional on $\boldsymbol{\beta}$ as

   $$z_i^{u*} = \ln(\lambda_1 U_i + z_i) - \ln(1 - U_i + \lambda_i(1 - z_i)),$$

   where U_i are independent Uniform random variables and $\ln \lambda_i = \boldsymbol{x}_i^T \boldsymbol{\beta}$.

   2.2 Generate the indicator variable r_i conditional on z_i^{u*} from

   $$\Pr(r_i = j | z_i^{u*}, \boldsymbol{\beta}) \propto \frac{w_j}{s_j} \exp\left(-\frac{1}{2}\left(\frac{z_i^{u*} - \ln \lambda_i}{s_j}\right)^2\right),$$

   and set $\omega_i = s^2_{r_i}$.

   2.3 Sample from $[\boldsymbol{\beta}|.]$ where $\boldsymbol{R} = (r_1, \ldots, r_{n_s})$, $\boldsymbol{\beta}|. \sim \mathcal{N}(\boldsymbol{\mu}_{\boldsymbol{\beta}},\ \boldsymbol{\Sigma}_{\boldsymbol{\beta}})$ where

   $$\boldsymbol{\mu}_{\boldsymbol{\beta}} = \boldsymbol{\Sigma}_{\boldsymbol{\beta}} \boldsymbol{X}^T \boldsymbol{S}_{\boldsymbol{\beta}}^{-1} \boldsymbol{z}^{u*} \text{ and}$$
   $$\boldsymbol{\Sigma}_{\boldsymbol{\beta}} = \left((\boldsymbol{\Sigma}^0_{\boldsymbol{\beta}})^{-1} + \boldsymbol{X}^T \boldsymbol{S}_{\boldsymbol{\beta}}^{-1} \boldsymbol{X}\right)^{-1}.$$

   $\boldsymbol{S}_{\boldsymbol{\beta}}$ is a diagonal matrix with diagonal entries $\omega_{1,\boldsymbol{\beta}}, \ldots, \omega_{n_s,\boldsymbol{\beta}}$.

   2.4 Generate the latent utilities y_{ij}^{u*}, $i = 1, \ldots, n^*$ conditional on $\boldsymbol{\alpha}$ as

   $$y_{ij}^{u*} = \ln(\lambda_{ij} U_i^* + y_{ij}) - \ln(1 - U_i^* + \lambda_{ij}(1 - y_{ij})),$$

where U_i^* are independent Uniform random variables and $\ln \lambda_{ij} = \boldsymbol{w}_{ij}^T \boldsymbol{\alpha}$. Here the indices (ij) index relates to those sites with $z_i = 1$.

2.5 Generate the indicator variable r_{ij}^* conditional on y_{ij}^{u*} from

$$\Pr(r_{ij}^* = j | y_{ij}^{u*}, \boldsymbol{\alpha}) \propto \frac{w_j}{s_j} \exp\left(-\frac{1}{2}\left(\frac{y_{ij}^{u*} - \ln \lambda_{ij}}{s_j}\right)^2\right),$$

and set $\omega_{ij} = s_{r_i}^2$.

2.6 Sample from $[\boldsymbol{\alpha}|.]$ where $\boldsymbol{R}^* = (r_{1,1}^*, \ldots, r_{n^*,K_{n^*}}^*)$, $\boldsymbol{\alpha}|. \sim \mathcal{N}(\boldsymbol{\mu}_{\boldsymbol{\alpha}}, \boldsymbol{\Sigma}_{\boldsymbol{\alpha}})$ where

$$\boldsymbol{\mu}_{\boldsymbol{\alpha}} = \boldsymbol{\Sigma}_{\boldsymbol{\alpha}} \tilde{\boldsymbol{W}}^T \boldsymbol{S}_{\boldsymbol{\alpha}}^{-1} \boldsymbol{y}^{u*} \text{ and}$$
$$\boldsymbol{\Sigma}_{\boldsymbol{\alpha}} = \left((\boldsymbol{\Sigma}_{\boldsymbol{\alpha}}^0)^{-1} + \tilde{\boldsymbol{W}}^T \boldsymbol{S}_{\boldsymbol{\alpha}}^{-1} \tilde{\boldsymbol{W}}\right)^{-1}.$$

$\boldsymbol{S}_{\boldsymbol{\alpha}}$ is a diagonal matrix with diagonal entries $\omega_{1,1,\boldsymbol{\alpha}}, \ldots, \omega_{n^*,K_{n^*},\boldsymbol{\alpha}}$. The matrix $\tilde{\boldsymbol{W}}$ relates to those entries in $\boldsymbol{W}$ associated with $z_i = 1$.

2.7 If the species is observed at location i, then $z_i = 1|. \sim \text{Bernoulli}(1)$ but if the species is not observed at location i, then

$$z_i = 1|. \sim \text{Bernoulli}\left(\frac{\psi_i \prod_j (1 - p_{ij})}{1 - \psi_i + \psi_i \prod_j (1 - p_{ij})}\right)$$

where ψ_i and p_{ij} are the occupancy and conditional detection probability of a species associated with each site and survey occasion.

1.3 Gibbs algorithm for the SSO model using Pólya-Gamma random variables.

Below we provide the full conditional distributions necessary to implement a Gibbs sampler for the SSO model when using Pólya-Gamma random variables. Here we assume that the prior distributions are $\boldsymbol{\alpha} \sim \mathcal{N}(\boldsymbol{\mu}_{\boldsymbol{\alpha}}^0,\ \boldsymbol{\Sigma}_{\boldsymbol{\alpha}}^0)$ and $\boldsymbol{\beta} \sim \mathcal{N}(\boldsymbol{\mu}_{\boldsymbol{\beta}}^0,\ \boldsymbol{\Sigma}_{\boldsymbol{\beta}}^0)$. Denote the prior precision matrices of $\boldsymbol{\alpha}$ and $\boldsymbol{\beta}$ as $\boldsymbol{\Lambda}_{\boldsymbol{\alpha}}^0$ and $\boldsymbol{\Lambda}_{\boldsymbol{\beta}}^0$.

1. Set starting values for $\boldsymbol{\alpha}$, $\boldsymbol{\beta}$ and $\boldsymbol{z}$.

2. Cycle:

   2.1 Generate $\omega_{i,\boldsymbol{\beta}}|. \sim \text{PG}(1, \boldsymbol{x}_i^T\boldsymbol{\beta})$.

   2.2 Sample from $[\boldsymbol{\beta}|.]$ where $\boldsymbol{\beta}|. \sim \mathcal{N}(\boldsymbol{\mu}_{\boldsymbol{\beta}},\ \boldsymbol{\Sigma}_{\boldsymbol{\beta}})$ with,

$$\begin{aligned}\boldsymbol{\mu}_{\boldsymbol{\beta}} &= \boldsymbol{\Sigma}_{\boldsymbol{\beta}}\left(\boldsymbol{X}^T(\boldsymbol{z} - 0.5\mathbf{1}_n) + \boldsymbol{\Lambda}_{\boldsymbol{\beta}}^0\boldsymbol{\mu}_{\boldsymbol{\beta}}^0\right) \text{ and}\\ \boldsymbol{\Sigma}_{\boldsymbol{\beta}} &= \left(\boldsymbol{\Lambda}_{\beta}^0 + \boldsymbol{X}^T\boldsymbol{S}_{\beta}\boldsymbol{X}\right)^{-1}.\end{aligned}$$

   $\boldsymbol{S}_{\boldsymbol{\beta}}$ is a diagonal matrix with diagonal entries $\omega_{1,\boldsymbol{\beta}}, \ldots, \omega_{n_s,\boldsymbol{\beta}}$.

   2.3 Generate $\omega_{ij,\boldsymbol{\alpha}}|. \sim \text{PG}(1, \boldsymbol{w}_{ij}^T\boldsymbol{\alpha})$.

   2.4 Sample from $[\boldsymbol{\alpha}|.]$ where $\boldsymbol{\alpha}|. \sim \mathcal{N}(\boldsymbol{\mu}_{\boldsymbol{\alpha}},\ \boldsymbol{\Sigma}_{\boldsymbol{\alpha}})$ with

$$\begin{aligned}\boldsymbol{\mu}_{\boldsymbol{\alpha}} &= \boldsymbol{\Sigma}_{\boldsymbol{\alpha}}\left(\tilde{\boldsymbol{W}}^T(\tilde{\boldsymbol{y}} - 0.5\mathbf{1}_{n^*}) + \boldsymbol{\Lambda}_{\boldsymbol{\alpha}}^0\boldsymbol{\mu}_{\boldsymbol{\alpha}}^0\right) \text{ and}\\ \boldsymbol{\Sigma}_{\boldsymbol{\alpha}} &= \left(\boldsymbol{\Lambda}_{\boldsymbol{\alpha}}^0 + \tilde{\boldsymbol{W}}^T\boldsymbol{S}_{\boldsymbol{\alpha}}\tilde{\boldsymbol{W}}\right)^{-1}.\end{aligned}$$

   $\boldsymbol{S}_{\boldsymbol{\alpha}}$ is a diagonal matrix with diagonal entries $\omega_{1,1,\boldsymbol{\alpha}}, \ldots, \omega_{n^*,K_{n^*},\boldsymbol{\alpha}}$. The matrices $\tilde{\boldsymbol{W}}$ and $\tilde{\boldsymbol{y}}$ relates to those entries in $\boldsymbol{W}$ and $\tilde{\boldsymbol{y}}$ associated with $z_i = 1$. The $\tilde{\boldsymbol{y}}$ vector is constructed by column stacking the entries of the observed detection/non-detection data $\boldsymbol{y}$.

   2.5 Sampling from $[z_i|.]$ is the same as in Appendix 1.2.

1.4 Gibbs algorithm for a spatial occupancy model using Pólya-Gamma random variables.

Denote the true occupancy variable as $\boldsymbol{z} = [\boldsymbol{z}^{(s)}, \boldsymbol{z}^{(s)'}]^T$ where $\boldsymbol{z}^{(s)}$ and $\boldsymbol{z}^{(s)'}$ represents the true occupancy variables at sites that were **surveyed** and **not surveyed** respectively. n represents the number of sites in the study (i.e. $n = \text{length}(\boldsymbol{z})$) while n_s is the number of sites surveyed. Let

$$\boldsymbol{X} = \begin{bmatrix} \boldsymbol{X}^{(s)} \\ \boldsymbol{X}^{(s)'} \end{bmatrix} \text{ and } \boldsymbol{K} = \begin{bmatrix} \boldsymbol{K}^{(s)} \\ \boldsymbol{K}^{(s)'} \end{bmatrix} \tag{1.4}$$

where both $\boldsymbol{X}^{(s)}$ and $\boldsymbol{K}^{(s)}$ have n_s rows.

Below we provide the full conditional distributions necessary for implementing a Gibbs sampler for a restricted spatial regression (RSR) occupancy model using Pólya-Gamma random variables. Here we assume that the prior distributions are $\boldsymbol{\alpha} \sim \mathcal{N}(\boldsymbol{\mu}_{\boldsymbol{\alpha}}^0,\ \boldsymbol{\Sigma}_{\boldsymbol{\alpha}}^0)$, $\boldsymbol{\beta} \sim \mathcal{N}(\boldsymbol{\mu}_{\boldsymbol{\beta}}^0,\ \boldsymbol{\Sigma}_{\boldsymbol{\beta}}^0)$, $\boldsymbol{\theta}|\tau \sim \mathcal{N}\left(\boldsymbol{0},\ \frac{1}{\tau}\boldsymbol{M}\right)$ and $\tau \sim \mathcal{G}(i_1,\ i_2)$. Denote the prior precision matrices of $\boldsymbol{\alpha}$ and $\boldsymbol{\beta}$ as $\boldsymbol{\Lambda}_{\boldsymbol{\alpha}}^0$ and $\boldsymbol{\Lambda}_{\boldsymbol{\beta}}^0$.

1. Set starting values for $\boldsymbol{\alpha}$, $\boldsymbol{\beta}$, $\boldsymbol{\theta}$ and $\boldsymbol{z}^{(s)}$.

2. Cycle:

   2.1 Generate $\omega_{i,\boldsymbol{\beta}}|. \sim \text{PG}(1, \boldsymbol{x}_i^T\boldsymbol{\beta} + \boldsymbol{k}_i^T\boldsymbol{\theta})$, for all $i = 1, \ldots, n_s$.

   2.2 Sample from $[\boldsymbol{\beta}|.]$ where $\boldsymbol{\beta}|. \sim \mathcal{N}\left(\boldsymbol{\mu}_{\boldsymbol{\beta}},\ \boldsymbol{\Sigma}_{\boldsymbol{\beta}}\right)$ with

$$\boldsymbol{\mu}_{\boldsymbol{\beta}} = \boldsymbol{\Sigma}_{\boldsymbol{\beta}}\left(\boldsymbol{X}^{(s)T}(\boldsymbol{z}^{(s)} - 0.5\boldsymbol{1}_{n_s} - \boldsymbol{S}_{\boldsymbol{\beta}}\boldsymbol{K}^{(s)}\boldsymbol{\theta}) + \boldsymbol{\Lambda}_{\boldsymbol{\beta}}^0\boldsymbol{\mu}_{\boldsymbol{\beta}}^0\right) \text{ and}$$
$$\boldsymbol{\Sigma}_{\boldsymbol{\beta}} = \left(\boldsymbol{\Lambda}_{\boldsymbol{\beta}}^0 + \boldsymbol{X}^{(s)T}\boldsymbol{S}_{\boldsymbol{\beta}}\boldsymbol{X}^{(s)}\right)^{-1}.$$

   $\boldsymbol{S}_{\boldsymbol{\beta}}$ is a diagonal matrix with diagonal entries $\omega_{1,\boldsymbol{\beta}}, \ldots, \omega_{n_s,\boldsymbol{\beta}}$.

   2.3 Sample from $[\boldsymbol{\theta}|.]$ where $\boldsymbol{\theta}|. \sim \mathcal{N}(\boldsymbol{\mu}_{\boldsymbol{\theta}}, \boldsymbol{\Sigma}_{\boldsymbol{\theta}})$ with

$$\boldsymbol{\mu}_{\boldsymbol{\theta}} = \boldsymbol{\Sigma}_{\theta}\boldsymbol{K}^{(s)T}\left(\boldsymbol{z}^{(s)} - 0.5\boldsymbol{1}_{n_s} - \boldsymbol{S}_{\boldsymbol{\beta}}\boldsymbol{X}^{(s)}\boldsymbol{\beta}\right)$$

$$\boldsymbol{\Sigma}_{\boldsymbol{\theta}} = \left(\tau \boldsymbol{M}^{-1} + \boldsymbol{K}^{(s)T} \boldsymbol{S}_{\boldsymbol{\beta}} \boldsymbol{K}^{(s)}\right)^{-1}.$$

2.4 Generate $\tau|. \sim \mathcal{G}\left(\frac{r}{2} + i_1,\ \frac{\boldsymbol{\theta}^T \boldsymbol{M}^{-1} \boldsymbol{\theta}}{2} + i_2\right)$.

2.5 Generate $\omega_{ij,\boldsymbol{\alpha}}|. \sim \text{PG}(1, \boldsymbol{w}_{ij}^T \boldsymbol{\alpha}),$ for all (ij) satisfying $\{z_i = 1\}$.

2.6 Sample from $[\boldsymbol{\alpha}|.]$ as done in Appendix 1.3.

2.7 (a) **For a surveyed site:** If the species is observed at location i, then $z_i = 1|. \sim$ Bernoulli(1) but if the species is not observed at location i, then

$$z_i = 1|. \sim \text{Bernoulli}\left(\frac{\psi_i \prod_j (1 - p_{ij})}{1 - \psi_i + \psi_i \prod_j (1 - p_{ij})}\right)$$

where ψ_i and p_{ij} are the occupancy and conditional detection probability of a species associated with each site and survey occasion.

2.7 (b) **For an unsurveyed site:** $z_i = 1|. \sim \text{Bernoulli}(\psi_i)$.

1.4.1 A brief derivation of the conditional distribution of the occupancy regression effects.

Take note that the conditional posterior distributions of $\boldsymbol{\beta}$ rely on the observation that $\psi^{z_i}(1 - \psi_i)^{1-z_i}$ can be rewritten as

$$\frac{\exp\left(\boldsymbol{x}_i^T \boldsymbol{\beta} + \boldsymbol{k}_i^T \boldsymbol{\theta}\right)^{z_i}}{1 + \exp\left(\boldsymbol{x}_i^T \boldsymbol{\beta} + \boldsymbol{k}_i^T \boldsymbol{\theta}\right)} = \exp(\kappa_i(\boldsymbol{x}_i^T \boldsymbol{\beta} + \boldsymbol{k}_i^T \boldsymbol{\theta})) \int \exp\left(-\frac{\omega_{i,\boldsymbol{\beta}}}{2}(\boldsymbol{x}_i^T \boldsymbol{\beta} + \boldsymbol{k}_i^T \boldsymbol{\theta})^2\right) p(\omega_{i,\boldsymbol{\beta}}|1, 0) d\omega_{i,\boldsymbol{\beta}}$$

where $p(\omega_{i,\boldsymbol{\beta}}|1, 0)$ is the probability density function of a Pólya-Gamma distribution with parameters 1 and 0. $\kappa_i = z_i - 0.5$ (Polson et al., 2013).

If we condition on $\boldsymbol{\omega}_{\boldsymbol{\beta}}$, the conditional posterior distribution of the occupancy regression effects are proportional to

$$[\boldsymbol{\beta}|.] \propto \pi(\boldsymbol{\beta}) \prod_{i=1}^{n} \exp(\kappa_i(\boldsymbol{x}_i^T \boldsymbol{\beta} + \boldsymbol{k}_i^T \boldsymbol{\theta})) \exp\left(-\frac{\omega_{i,\boldsymbol{\beta}}}{2}(\boldsymbol{x}_i^T \boldsymbol{\beta} + \boldsymbol{k}_i^T \boldsymbol{\theta})^2\right)$$

and after completing the square in terms of $\boldsymbol{\beta}$ we obtain the conditional posterior distribution as reported in the above algorithm.

The conditional posterior distribution of $\boldsymbol{\alpha}$ and $\boldsymbol{\theta}$ both use the same manipulation of the Bernoulli likelihood.

1.5 Summary statistics of the climate covariates.

- The variables *GDD0* and *GDD5* relate to annual thermal sums above 0 and 5 degrees centigrade respectively.
- The variables *MTCO* and *MTWA* relate to the mean temperature of the coldest and warmest month respectively.
- The variable *AETPET* is the ratio of potential to realized evapotranspiration.
- The variables *DryInt* and *WetInt* relates to the intensity of the dry and wet season respectively.

Table S2. Lower triangle of the correlation matrix of the climate variables.

	GDD0	GDD5	MTCO	MTWA	AETPET	WetInt	DryInt
GDD0	1						
GDD5	1.00	1					
MTCO	**0.86**	**0.86**	1				
MTWA	**0.85**	**0.85**	0.50	1			
AETPET	-0.27	-0.27	0.05	**-0.61**	1		
WetInt	-0.24	-0.23	0.11	-0.51	**0.74**	1	
DryInt	-0.34	-0.34	-0.00	**-0.65**	**0.93**	**0.62**	1

Table S3. Variance inflation factors of the climate variables.

Variables	GDD0	GDD5	MTCO	MTWA	AETPET	WetInt	DryInt
VIF	3 393.35	3 371.0	25.85	32.65	16.74	4.44	10.40

Table S4. Summary statistics of the principal components of the climate variables.

	PC1	PC2	PC3	PC4	PC5	PC6	PC7
Standard deviation	2.03	1.48	0.64	0.45	0.24	0.099	0.012
Cumulative Proportion	0.59	0.90	0.96	0.99	0.999	0.9999	1

1.6 Plots of the climate variables and the associated principal components.

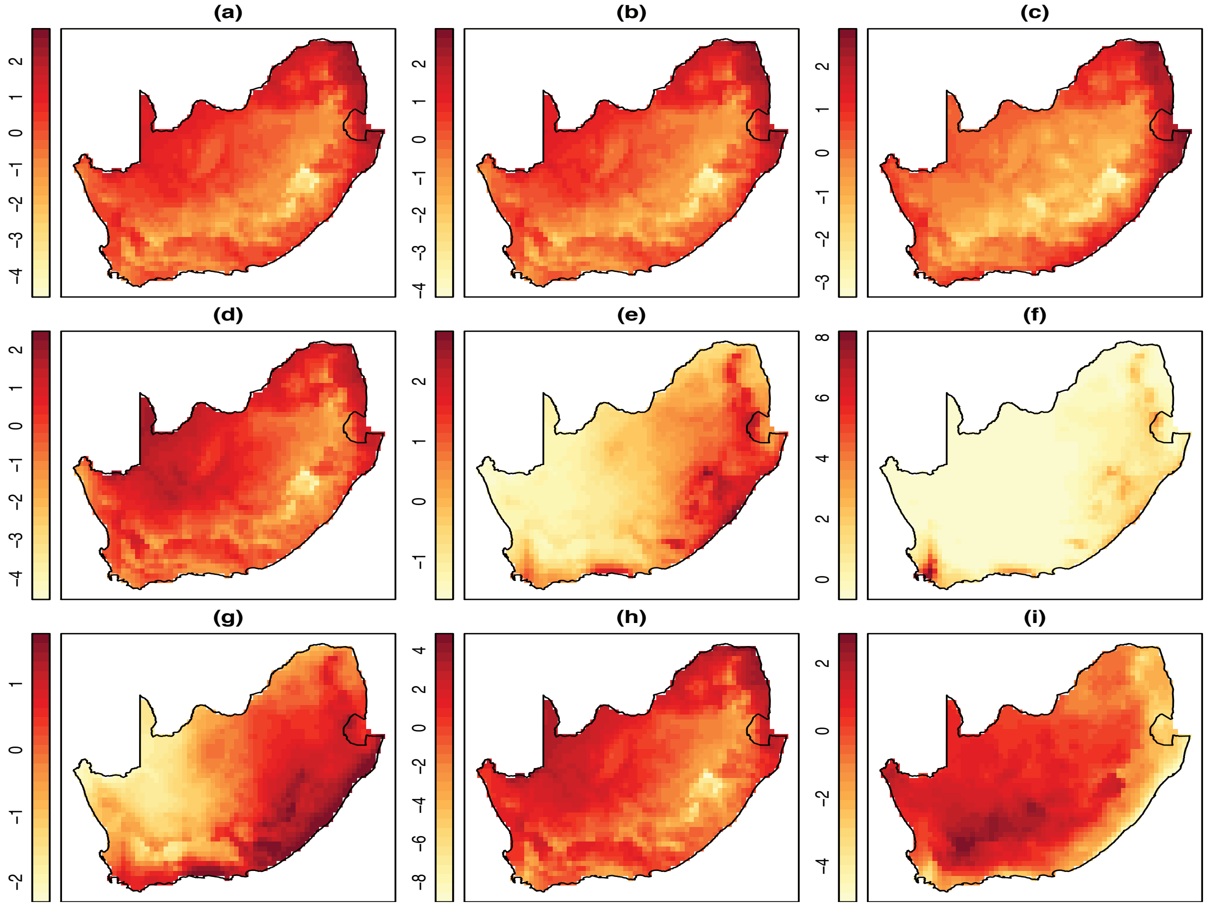

Figure S5. The climate variables as well as the first two principal components of the climate variables ((a) = GDD0, (b) = GDD5, (c)= MTCO, (d)= MTWA, (e) = AETPET, (f)= WetInt, (g) = DryInt, (h) = 1st principal component, (i) = 2nd principal component).

1.7 A worked example using simulated spatial data

Below we provide a worked example of how to fit the RSR model (in R) using *stocc*, *Stan* as well as *Rcppocc*. Ensure that you have installed the following R packages: *rstan*, *stocc* and *Rcppocc*. Installation instructions for *Rcppocc* can be found at `https://github.com/AllanClark/Rcppocc`.

We model the detection process using one covariate and the occupancy process using two covariates. In both cases, the intercept is included in the occupancy and detection processes. 188 spatial random effects are included in the spatial occupancy process. The same prior distributions as specified in the main part of the manuscript are used below.

Load the R data (contained in the *Rcppocc* package) file as well as some R packages.

```
require(stocc)
require(Rcppocc)

require(rstan)
rstan_options(auto_write = TRUE)


data(SpatSimData)
```

Create some R objects that will be used below.

```
xycords <- SimTable$xycords
psi <- SimTable$psi
z <- SimTable$z
alpha <- SimTable$alpha

Nvisits_surveyed <- SimTable$Nvisits_surveyed

Xmat <- SimTable$Xmat
Minv <- SimTable$Minv
Q <- SimTable$Q
Kmat <- SimTable$Kmat
Wmat <- SimTable$Wmat

pij <- SimTable$pij
Ysim <- SimTable$Ysim

nsites <- SimTable$nsites
Num_surveys <- SimTable$Num_surveys
N_maxvisits <- SimTable$N_maxvisits
```

The *stocc* fit

Fit the probit model using *stocc*.

```
#bundle data into the correct format for stocc
#occupancy dataframe at the subset of sites where surveys were performed
surveyIndex <- 1:Num_surveys #surveyIndex are the ids of the surveyed locations
siteIndex <- 1:nsites #ids for all sites considered

Site.Data <- as.data.frame( cbind( siteIndex, xycords, Xmat ) )
colnames(Site.Data)<- c("SiteName", "Longitude", "Latitude", "PC1", "PC2")

#detection dataframe - only at surveyed locations
PAdata <- na.omit(c(t(Ysim))) #presence-absence data
SiteNamereps <- rep(surveyIndex, Nvisits_surveyed) #the site names
nspp <- na.omit(c(t(Wmat))) #detection covariate
Longitudereps <- rep(xycords[surveyIndex,1], Nvisits_surveyed)
Latitudereps <- rep(xycords[surveyIndex,2], Nvisits_surveyed)

#at surveyed locations
Visit.data <- data.frame(SiteNamereps, Longitudereps, Latitudereps, nspp, PAdata)
colnames(Visit.data) <- c("SiteName", "Longitude", "Latitude", "nspp", "PAdata")

Names <- list(visit = list(site = "SiteName", obs = "PAdata"),
site = list(site = "SiteName", coords = c("Longitude", "Latitude")) )

Make.so.data <- make.so.data(visit.data = Visit.data,
site.data = Site.Data, names = Names)
```

Set prior distributions and some simulation settings.

```
#Set the prior distributions used
nalphas<-2
nbetas<-3

beta_m<-matrix( rep(0,nbetas), ncol=1)
sigma_inv_beta_p<-diag(nbetas)/1000 #prior inverse covariance for beta

alpha_m<-matrix( rep(0,nalphas), ncol=1)
sigma_inv_alpha_p<-diag(nalphas)/1000 #prior inverse covariance for alpha

numSpatre<-188

#mcmc settings
#These should be increased and are simply set to make it fairly fast to run!
nburnin <- 10000
niter <- 10000
nthin <- 1
```

The spatial.occupancy call.

```
spat_probit <- spatial.occupancy(detection.model= ~ nspp,
occupancy.model= ~ PC1 + PC2, spatial.model = list(model = "rsr",
threshold = 0.36, moran.cut = numSpatre, rho=1), so.data = Make.so.data,
prior = list(a.tau = 0.5, b.tau = 0.0005,
Q.b=sigma_inv_alpha_p, mu.b = alpha_m,
Q.g=sigma_inv_beta_p, mu.g = beta_m),
control = list(burnin = nburnin, iter = niter, thin = nthin))
```

The *Rcppocc* fit

```
spat_logit <- occSPATlogit(detection.model= ~ nspp, occupancy.model= ~ PC1 + PC2,
spatial.model = list(threshold = 0.36, moran.cut = numSpatre, rho=1),
so.data = Make.so.data,
prior = list(a.tau = 0.5, b.tau = 0.0005,
tau=1,
Q.d=sigma_inv_alpha_p, mu.d = alpha_m,
Q.o=sigma_inv_beta_p, mu.o = beta_m),
control = list(ndraws =niter, percent_burn_in = 0.5))
```

The *Stan* fit

```
#see https://github.com/stan-dev/example-models/blob/master/misc/ecology
#/occupancy/occupancy.stan  - for simple occupancy code

#see http://mc-stan.org/users/documentation/case-studies/mbjoseph-CARStan.html
# - for sparse icar model

SSS03 <- "
functions {
   //note the '188' has been hard coded.
   //this can be passed as an argument as well.

   real theta_lpdf(vector theta, real tau, matrix Minv){
   return 0.5*( 188*log(tau) - tau*quad_form(Minv, theta) );
   }
}

data {
   //note the '188' has been hard coded.

   int<lower=1> nsites;
   int N_maxvisits; //maximum number of survey visits
   int V[nsites]; //number of visits

   int<lower=0,upper=1> y[nsites, N_maxvisits]; //presence absence data
```

```
  matrix[nsites, 3] Xmat; // X variable
  matrix[nsites, 188] Kmat; // K variable
  real Wmat[nsites, N_maxvisits]; // W matrix - detection covariates
  matrix[188, 188] Minv; //K.t*Q*K
}

parameters {
  //note the '188' has been hard coded.

  vector[3] beta;  //occupancy slope params
  vector[2] alpha;  //detection slope params
  vector[188] theta;
  real<lower = 0> tau;
}

transformed parameters {
  vector[nsites] psi; // prob of of occurrence
  real pij[nsites, N_maxvisits]; // prob of detection

  //calculate psi_i and pij
  for (isite in 1:nsites){
    //here Xmat[isite]*beta takes the 'isite'th row of Xmat and
    //multiplies it by beta
    psi[isite] = inv_logit( Xmat[isite]*beta + Kmat[isite]*theta  );

    for (ivisit in 1:N_maxvisits){
      pij[isite, ivisit] = inv_logit( alpha[1] +
      Wmat[isite, ivisit]*alpha[2] );
    }
  }
}

model {
  vector[nsites] log_psi;
  vector[nsites] log1m_psi;

  for (isite in 1:nsites) {
    log_psi[isite] = log(psi[isite]);
    log1m_psi[isite] = log1m(psi[isite]);
  }

  // priors
  tau ~ gamma(0.5, 0.005);
  theta ~ theta_lpdf(tau, Minv);
  alpha ~ normal(0, 1000);
  beta ~ normal(0, 1000);

  // likelihood
  for (isite in 1:nsites) {

    if (sum(y[isite, 1:V[isite]]) > 0){
      target += log_psi[isite] +
```

```
          bernoulli_lpmf(y[isite, 1:V[isite]]|pij[isite,1:V[isite]]) ;
       }else {
          target += log_sum_exp(log_psi[isite] +
          bernoulli_lpmf(y[isite, 1:V[isite]]|pij[isite, 1:V[isite]]),
          log1m_psi[isite]);
       }
    }//end likelihood contribution
}
"
```

Compile the model and run it.

```
Model_code_covocc3 <- stan_model(model_code = SSSO3)

V <- Nvisits_surveyed

#Note redefinition of some objects!

y <- Ysim
y[is.na(y)] <- 0 #replace all NA elements of y with a number. Stan does not allow NA's
Wmat[is.na(Wmat)] <- 0

Xmat <- cbind(1, Xmat[1:Num_surveys,]) #in the Stan code the intercept is included
Kmat <- Kmat[1:Num_surveys,]
nsites <- NROW(Xmat)

stanfit3 <- sampling(Model_code_covocc3, data=c("nsites","N_maxvisits","V","y",
"Xmat", "Kmat", "Wmat","Minv"), iter = niter, chains = 1, warmup = nburnin,
thin = 1, verbose=F)
```

1.8 Data Accessibility:

All data files will be archived in Dryad.

Bibliography

Andrews, D. F. and Mallows, C. L. (1974). Scale mixtures of normal distributions. *Journal of the Royal Statistical Society. Series B (Methodological)*, 36(1):99–102.

Balakrishnan, N., editor (1992). *Handbook of the logistic distribution*. Marcel Dekker, New York.

Carpenter, B., Gelman, A., Hoffman, M. D., Lee, D., Goodrich, B., Betancourt, M., Brubaker, M., Guo, J., Li, P., and Riddell, A. (2017). Stan: A probabilistic programming language. *Journal of Statistical Software*, 76(1):1–32.

Clark, A. E., Altwegg, R., and Ormerod, J. T. (2016). A variational Bayes approach to the analysis of occupancy models. *PLoS ONE*, 11(2):e0148966.

Eddelbuettel, D. and Francois, R. (2011). Rcpp: Seamless R and C++ integration. *Journal of Statistical Software*, 40(8):1–18.

Frühwirth-Schnatter, S. and Frühwirth, R. (2007). Auxiliary mixture sampling with applications to logistic models. *Computational Statistics & Data Analysis*, 51(7):3509–3528.

Frühwirth-Schnatter, S. and Frühwirth, R. (2010). Data augmentation and MCMC for binary and multinomial logit models. In Kneib, T. and Tutz, G., editors, *Statistical Modelling and Regression Structures*, pages 111–132. Physica-Verlag, Heidelberg.

Holmes, C. C. and Held, L. (2006). Bayesian auxiliary variable models for binary and multinomial regression. *Bayesian Analysis*, 1(1):145–168.

Kellner, K. (2014). jagsui: Run JAGS (specifically, libjags) from R; an alternative user interface for rjags. *R package version*, 1.

Link, W. A. and Eaton, M. J. (2012). On thinning of chains in MCMC. *Methods in Ecology and Evolution*, 3(1):112–115.

Mcfadden, D. (1974). Conditional logit analysis of qualitative choice behavior. In Zarembka, P., editor, *Frontiers in Econometrics*, pages 105–142. Academic Press, New York.

Monnahan, C. C., Thorson, J. T., and Branch, T. A. (2017). Faster estimation of bayesian models in ecology using Hamiltonian Monte Carlo. *Methods in Ecology and Evolution*, 8(3):339–348.

Plummer, M. (2003). JAGS: A program for analysis of Bayesian graphical models using Gibbs sampling. In Hornik, K., L. F. and Zeileis, A., editors, *Proceedings of the 3rd international workshop on distributed statistical computing*. Technische Universit at Wien, Vienna, Austria.

Plummer, M., Best, N., Cowles, K., and Vines, K. (2006). CODA: Convergence Diagnosis and Output Analysis for MCMC. *R News*, 6(1):7–11.

Polson, N. G., Scott, J. G., and Windle, J. (2013). Bayesian inference for logistic models using Pólya-Gamma latent variables. *Journal of the American Statistical Association*, 108(504):1339–1349.

R Core Team (2014). *R: A Language and Environment for Statistical Computing*. R Foundation for Statistical Computing, Vienna, Austria.

Robert, C. and Casella, G. (1999). *Monte Carlo Statistical Methods*. Springer-Verlag, New York.

Sanderson, C. and Curtin, R. (2016). Armadillo: a template-based C++ library for linear algebra. *Journal of Open Source Software*, 1(2):26.

Windle, J., Polson, N., and Scott, J. (2013). BayesLogit: Bayesian logistic regression. *URL http://cran. r-project. org/web/packages/BayesLogit/index. html. R package version 0.2-4*.
