# Supplementary material for: Efficient Bayesian analysis of occupancy models with logit link functions
Source: Ecol Evol. 2019 Feb 5;9(2):756–68. doi: 10.1002/ece3.4850 (PMC6362608; doi:10.1002/ece3.4850)
Supplement: Supplementary file 1 [file ECE3-9-756-s001.zip › SuppReferences.rtf]

\begin{thebibliography}{}\bibitem[Andrews and Mallows, 1974]{andrews1974scale}Andrews, D.~F. and Mallows, C.~L. (1974).\newblock Scale mixtures of normal distributions.\newblock {\em Journal of the Royal Statistical Society. Series B  (Methodological)}, 36(1):99--102.\bibitem[Balakrishnan, 1992]{balakrishnan1992handbook}Balakrishnan, N., editor (1992).\newblock {\em Handbook of the logistic distribution}.\newblock Marcel Dekker, New York.\bibitem[Carpenter et~al., 2017]{carpenter2017stan}Carpenter, B., Gelman, A., Hoffman, M.~D., Lee, D., Goodrich, B., Betancourt,  M., Brubaker, M., Guo, J., Li, P., and Riddell, A. (2017).\newblock Stan: A probabilistic programming language.\newblock {\em Journal of Statistical Software}, 76(1):1--32.\bibitem[Clark et~al., 2016]{clark2016variational}Clark, A.~E., Altwegg, R., and Ormerod, J.~T. (2016).\newblock A variational {B}ayes approach to the analysis of occupancy models.\newblock {\em PLoS ONE}, 11(2):e0148966.\bibitem[Eddelbuettel and Francois, 2011]{Rcpp}Eddelbuettel, D. and Francois, R. (2011).\newblock {Rcpp: {S}eamless R and C++ integration}.\newblock {\em Journal of Statistical Software}, 40(8):1--18.\bibitem[Fr{\"u}hwirth-Schnatter and Fr{\"u}hwirth,  2007]{fruhwirth2007auxiliary}Fr{\"u}hwirth-Schnatter, S. and Fr{\"u}hwirth, R. (2007).\newblock Auxiliary mixture sampling with applications to logistic models.\newblock {\em Computational Statistics \& Data Analysis}, 51(7):3509--3528.\bibitem[Fr{\"u}hwirth-Schnatter and Fr{\"u}hwirth, 2010]{fruhwirth2010data}Fr{\"u}hwirth-Schnatter, S. and Fr{\"u}hwirth, R. (2010).\newblock {Data augmentation and MCMC for binary and multinomial logit models}.\newblock In Kneib, T. and Tutz, G., editors, {\em Statistical Modelling and  Regression Structures}, pages 111--132. Physica-Verlag, Heidelberg.\bibitem[Holmes and Held, 2006]{holmes2006bayesian}Holmes, C.~C. and Held, L. (2006).\newblock Bayesian auxiliary variable models for binary and multinomial  regression.\newblock {\em Bayesian {A}nalysis}, 1(1):145--168.\bibitem[Kellner, 2014]{kellner2014jagsui}Kellner, K. (2014).\newblock jagsui: Run {JAGS} (specifically, libjags) from {R}; an alternative  user interface for rjags.\newblock {\em R package version}, 1.\bibitem[Link and Eaton, 2012]{link2012thinning}Link, W.~A. and Eaton, M.~J. (2012).\newblock {On thinning of chains in MCMC}.\newblock {\em Methods in Ecology and Evolution}, 3(1):112--115.\bibitem[Mcfadden, 1974]{mcfadden1974conditional}Mcfadden, D. (1974).\newblock Conditional logit analysis of qualitative choice behavior.\newblock In Zarembka, P., editor, {\em Frontiers in Econometrics}, pages  105--142. Academic Press, New York.\bibitem[Monnahan et~al., 2017]{monnahan2017faster}Monnahan, C.~C., Thorson, J.~T., and Branch, T.~A. (2017).\newblock Faster estimation of bayesian models in ecology using {H}amiltonian  {M}onte {C}arlo.\newblock {\em Methods in Ecology and Evolution}, 8(3):339--348.\bibitem[Plummer, 2003]{plummer2003jags}Plummer, M. (2003).\newblock {JAGS: A program for analysis of Bayesian graphical models using  Gibbs sampling}.\newblock In Hornik, K., L.~F. and Zeileis, A., editors, {\em Proceedings of  the 3rd international workshop on distributed statistical computing}.  Technische Universit at Wien, Vienna, Austria.\bibitem[Plummer et~al., 2006]{coda}Plummer, M., Best, N., Cowles, K., and Vines, K. (2006).\newblock {CODA}: Convergence {D}iagnosis and {O}utput {A}nalysis for {MCMC}.\newblock {\em R News}, 6(1):7--11.\bibitem[Polson et~al., 2013]{polson2013bayesian}Polson, N.~G., Scott, J.~G., and Windle, J. (2013).\newblock {Bayesian inference for logistic models using P{\'o}lya-Gamma latent  variables}.\newblock {\em Journal of the American Statistical Association},  108(504):1339--1349.\bibitem[{R Core Team}, 2014]{Rref}{R Core Team} (2014).\newblock {\em R: {A} {L}anguage and {E}nvironment for {S}tatistical  {C}omputing}.\newblock R Foundation for Statistical Computing, Vienna, Austria.\bibitem[Robert and Casella, 1999]{robert1999monte}Robert, C. and Casella, G. (1999).\newblock {\em {Monte {C}arlo Statistical Methods}}.\newblock Springer-Verlag, New York.\bibitem[Sanderson and Curtin, 2016]{sanderson2016armadillo}Sanderson, C. and Curtin, R. (2016).\newblock {Armadillo: a template-based C++ library for linear algebra}.\newblock {\em Journal of Open Source Software}, 1(2):26.\bibitem[Windle et~al., 2013]{windle2013bayeslogit}Windle, J., Polson, N., and Scott, J. (2013).\newblock {BayesLogit: {B}ayesian logistic regression}.\newblock {\em URL http://cran. r-project. org/web/packages/BayesLogit/index.  html. R package version 0.2-4}.\end{thebibliography}
